# Supplementary material for: Population structure and migration in the Eastern Highlands of Papua New Guinea, a region impacted by the kuru epidemic
Source: Am J Hum Genet. 2024 Mar 19;111(4):668–79. doi: 10.1016/j.ajhg.2024.02.011 (PMC11023820; doi:10.1016/j.ajhg.2024.02.011)
Supplement: Document S1. Figures S1–S9 and Tables S1–S3, S5, and S6 [file mmc1.pdf]

The American Journal of Human Genetics, Volume *111*

Supplemental information

Population structure and migration in the Eastern Highlands of Papua New Guinea, a region impacted by the kuru epidemic

Liam Quinn, Jerome Whitfield, Michael P. Alpers, Tracy Campbell, Holger Hummerich, William Pomat, Peter Siba, George Koki, Ida Moltke, John Collinge, Garrett Hellenthal, and Simon Mead

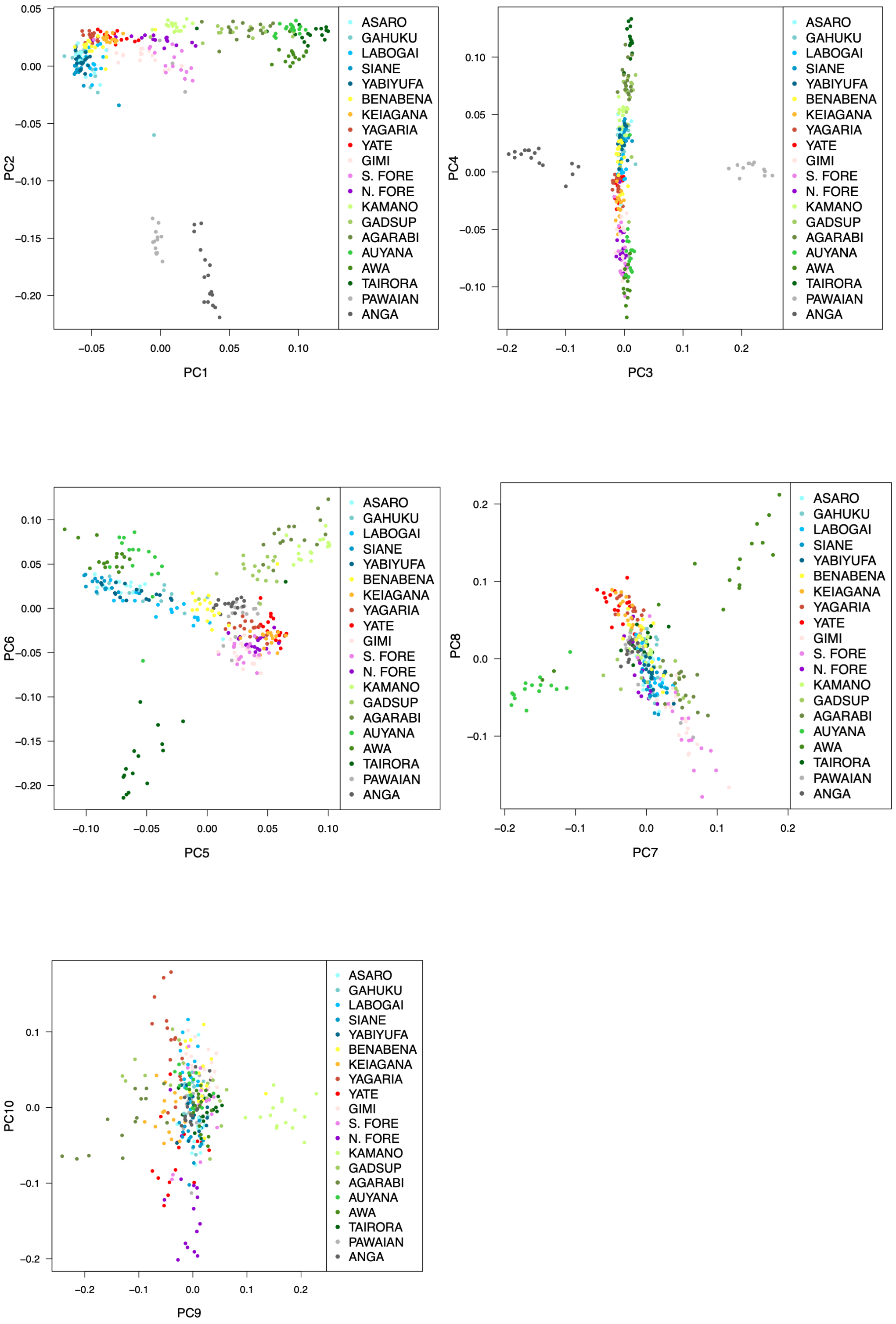

Figure S1 - Principal Components 1-10 of 320 individuals from 20 EHPNG linguistic groups

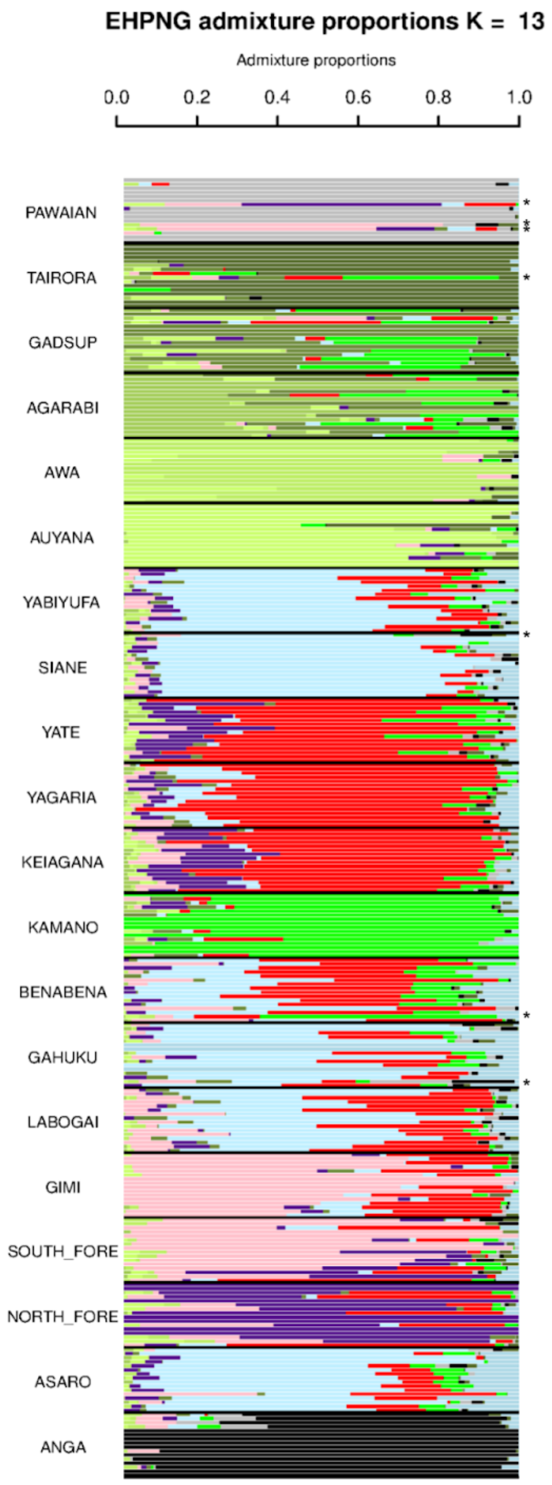
EHPNG admixture proportions K = 13
Admixture proportions
0.0
0.2
0.4
0.6
0.8
1.0
PAWAIAN
TAIRORA
GADSUP
AGARABI
AWA
AUYANA
YABIYUFA
SIANE
YATE
YAGARIA
KEIAGANA
KAMANO
BENABENA
GAHUKU
LABOGAI
GIMI
SOUTH_FORE
NORTH_FORE
ASARO
ANGA

Figure S2 - ADMIXTURE analysis of 320 individuals from 20 EHPNG linguistic groups for K=13. Individuals with * label in above ADMIXTURE profile are those individuals highlighted in Figure 1a and Figure 1b as potential migrants.

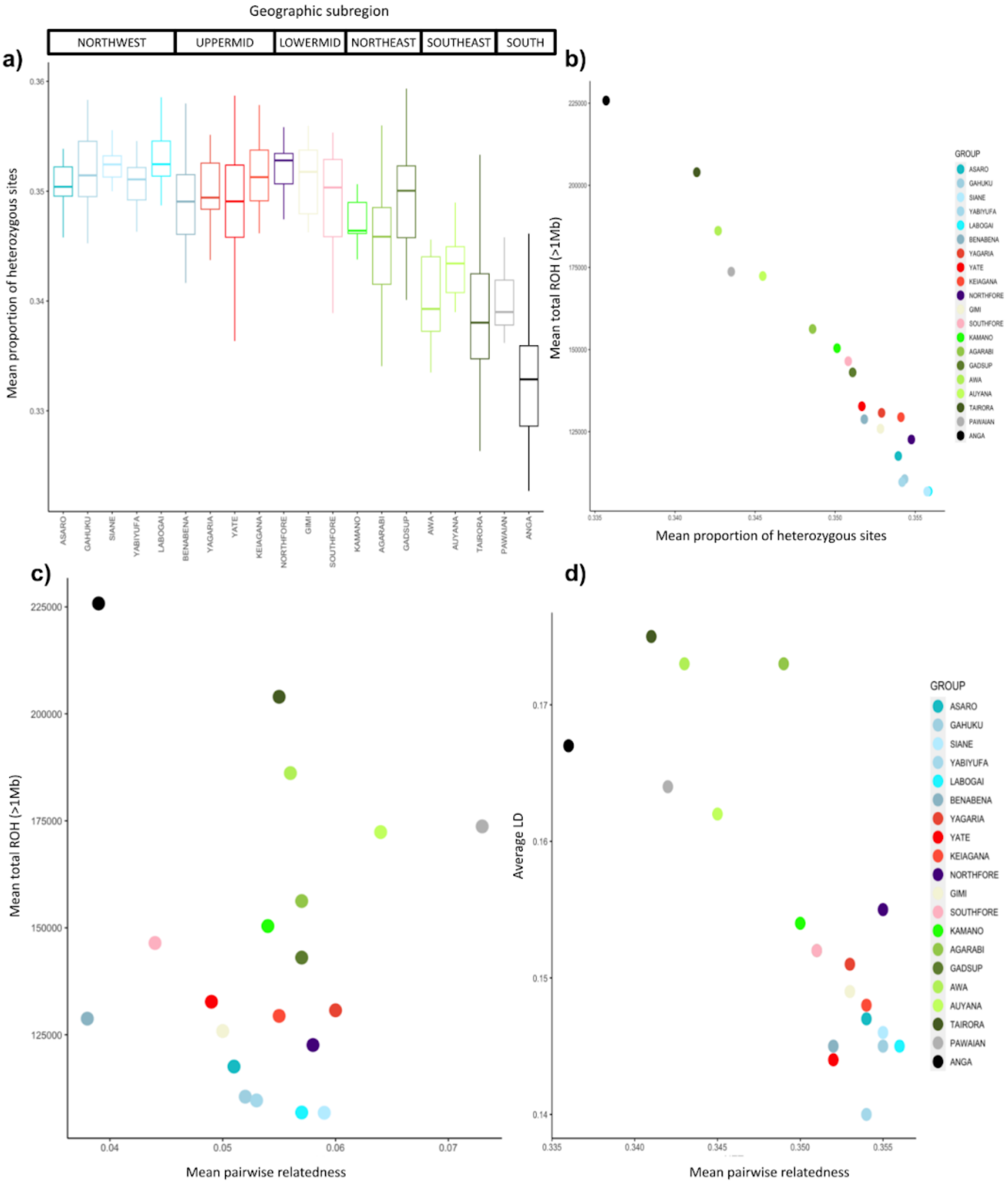

Figure S3 - a) Boxplot of mean proportion of heterozygous sites by geographic sublocation. b) Mean total length of ROH versus proportion of heterozygous sites. c) Mean total length of ROH versus mean pairwise relatedness. d) Mean average pairwise relatedness versus mean linkage disequilibrium. Plots are taken from analysis of *Linguistic Group Analysis* dataset.

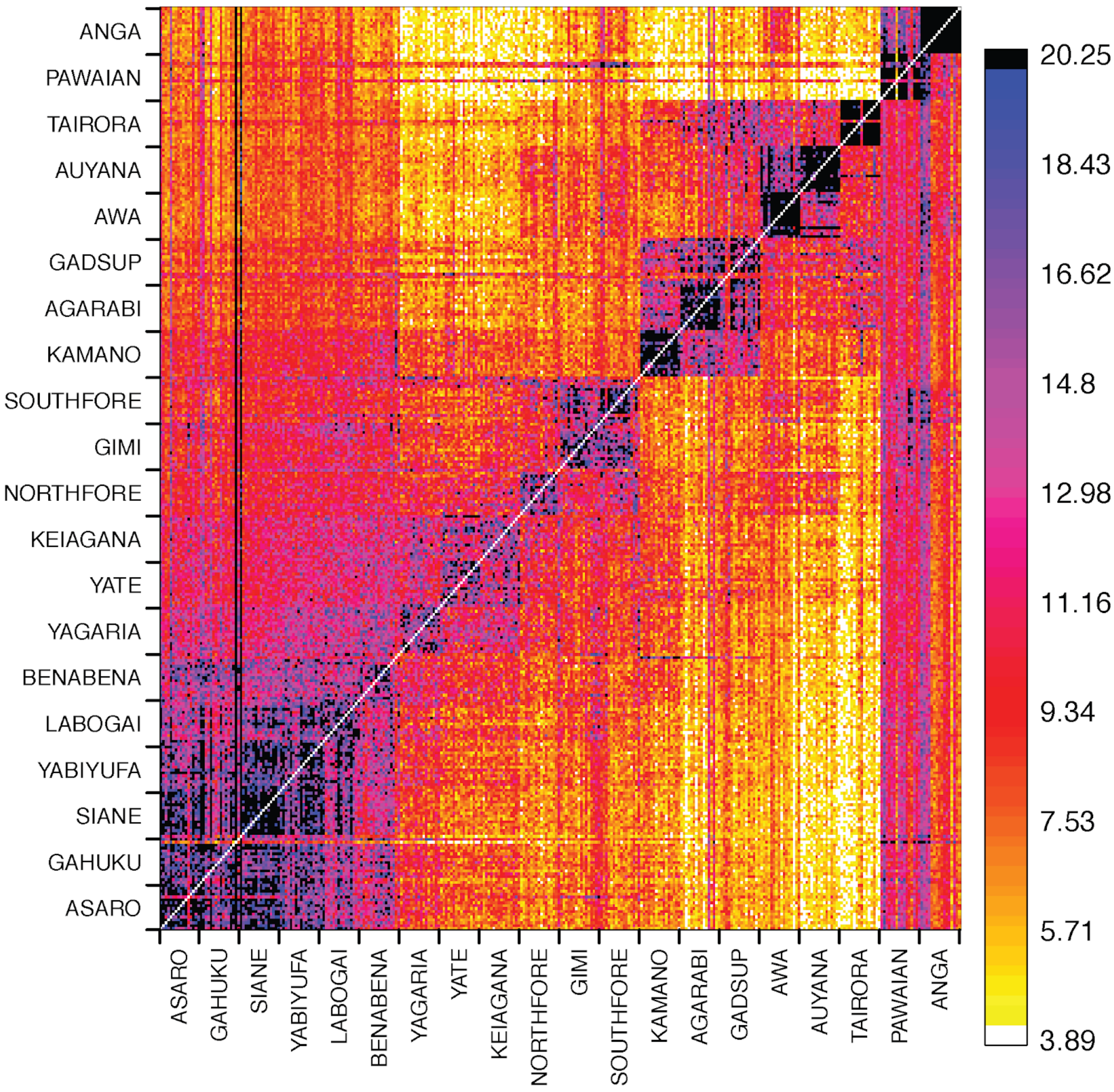

Figure S4 - CP heatmap of 320 individuals from 20 EHPNG linguistic groups.

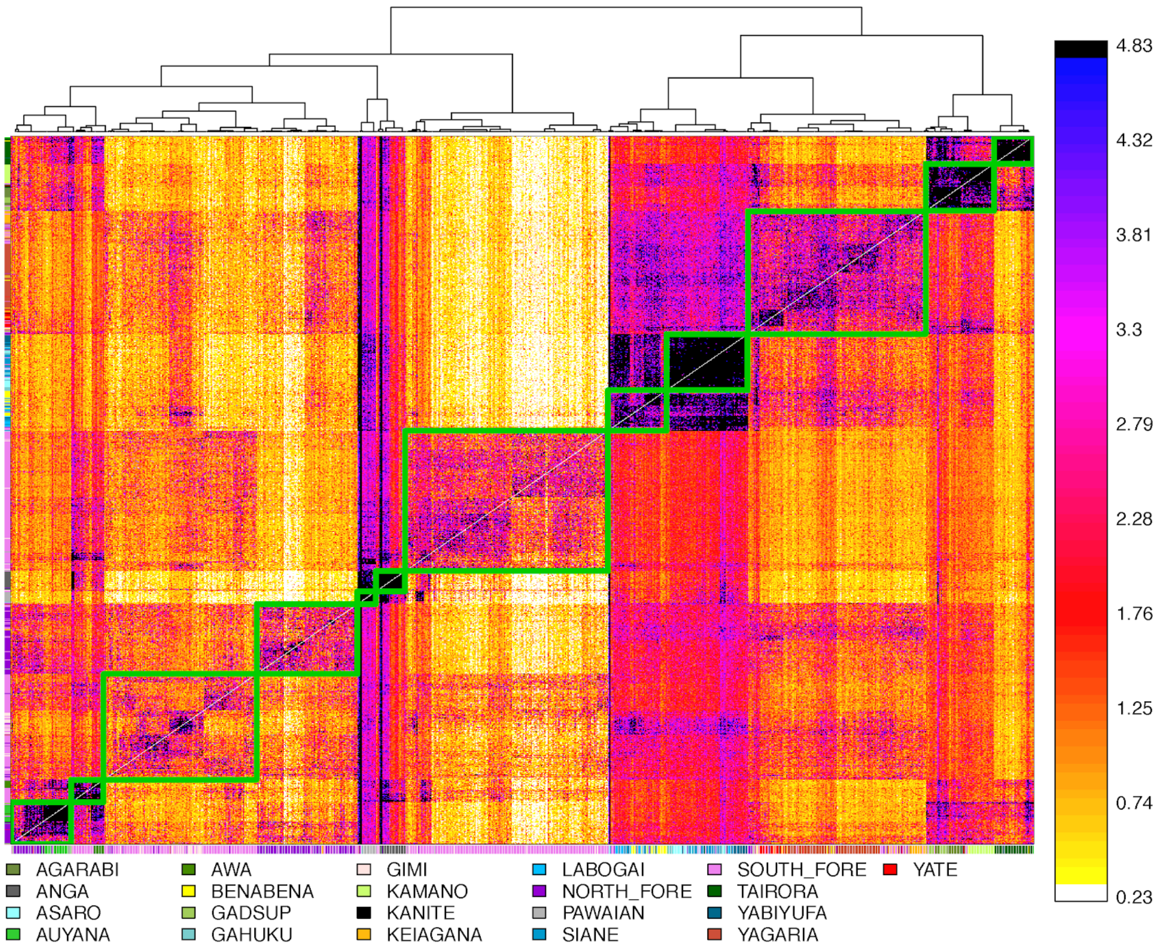

Figure S5 – CP heatmap of 943 individuals from 21 EHPNG Ethno-Linguistic Groups placed into 12 discrete clusters (green boxes on heatmap) as defined by FS.

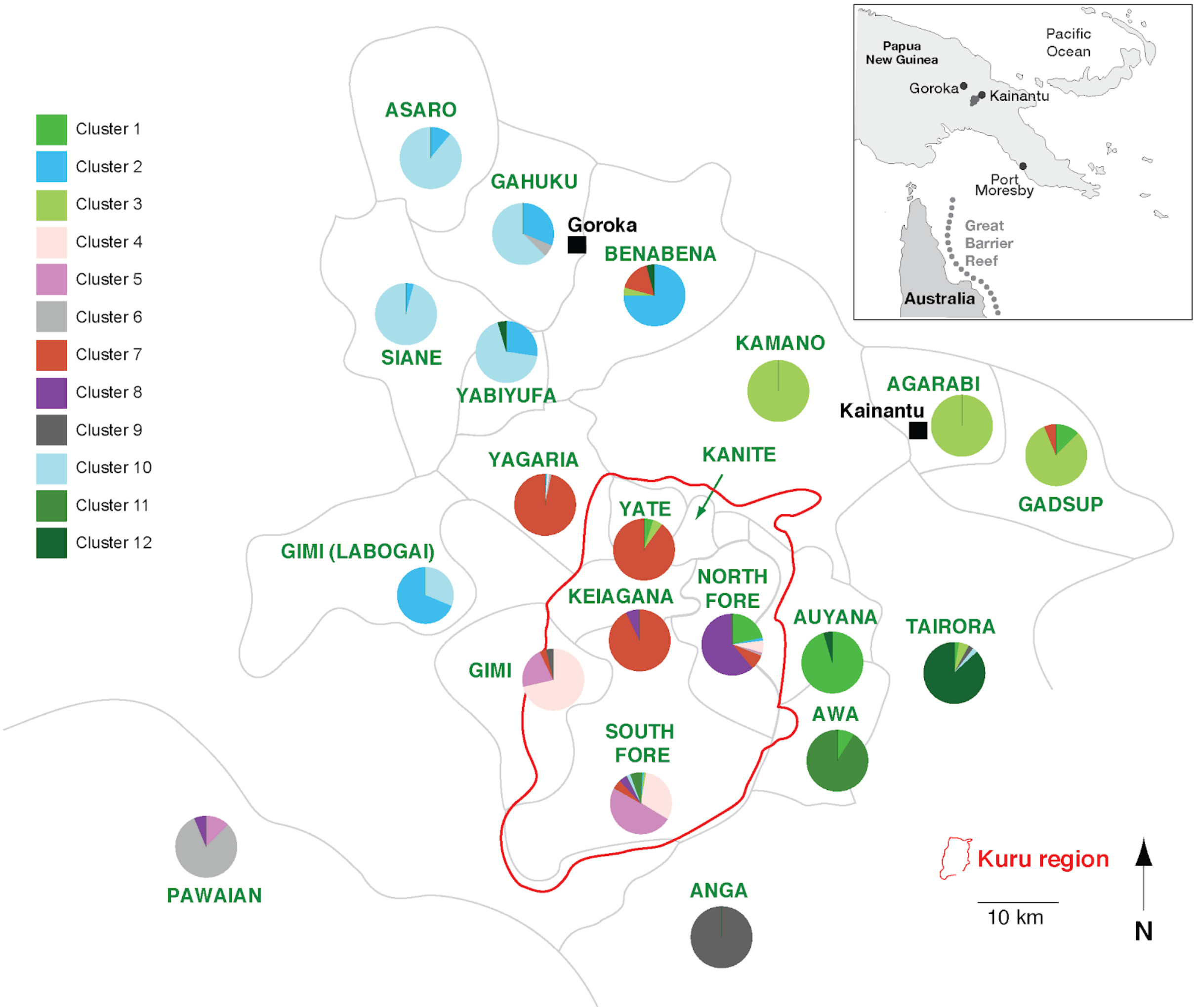

Figure S6 - Geographical representation of FS clustering (K=12) of individuals from *Village Analysis* CP output of Figure S5.

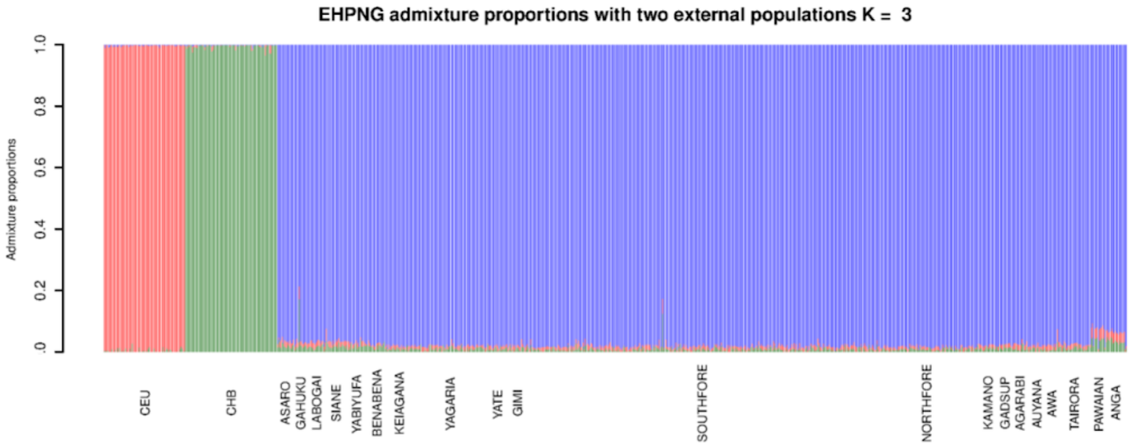

Figure S7 – ADMIXTURE analysis of 943 EHPNG individuals from 21 linguistic groups with 1000 genomes project populations CEU and YRI.

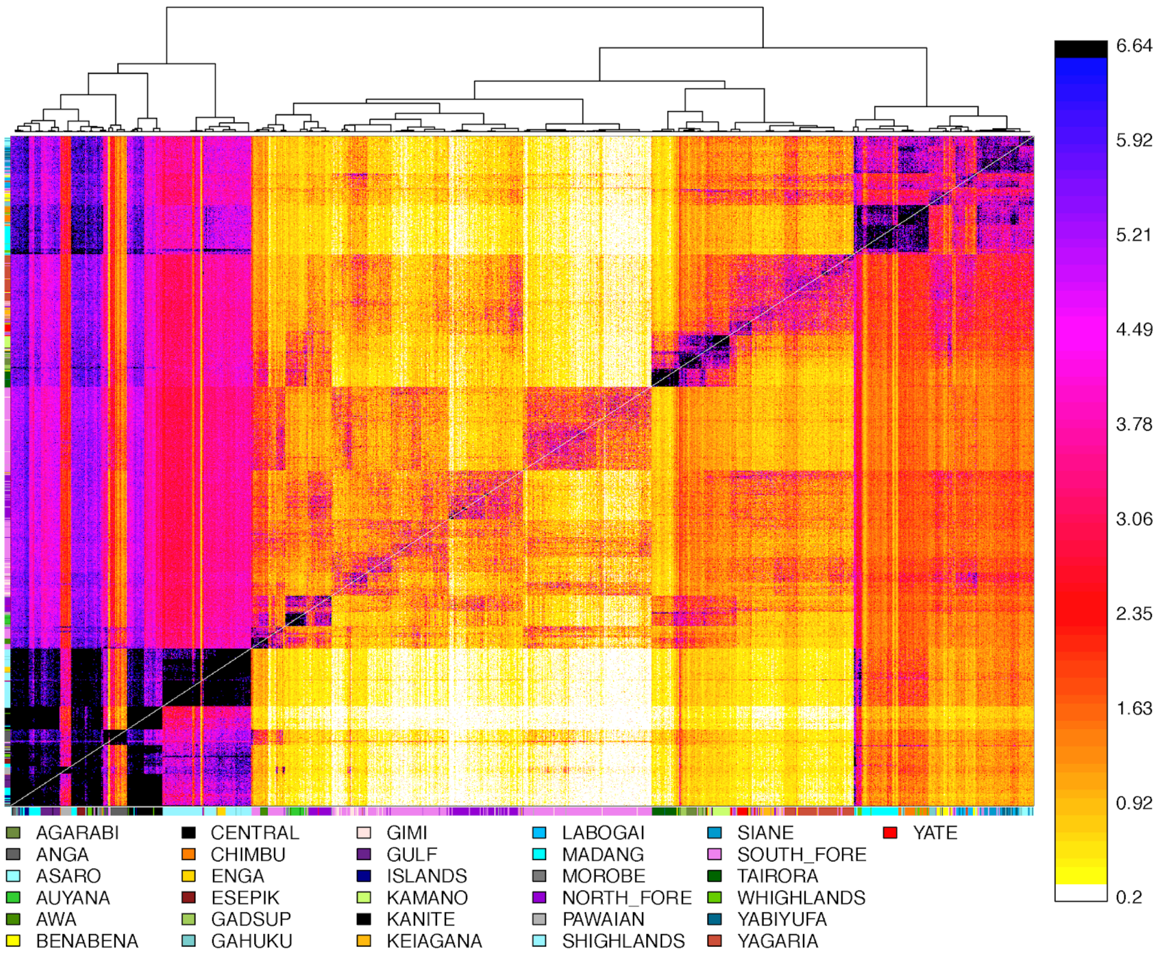

Figure S8 –CP heatmap of 1293 individuals, 943 from 21 EHPNG linguistic groups and 350 individuals from other PNG regions.

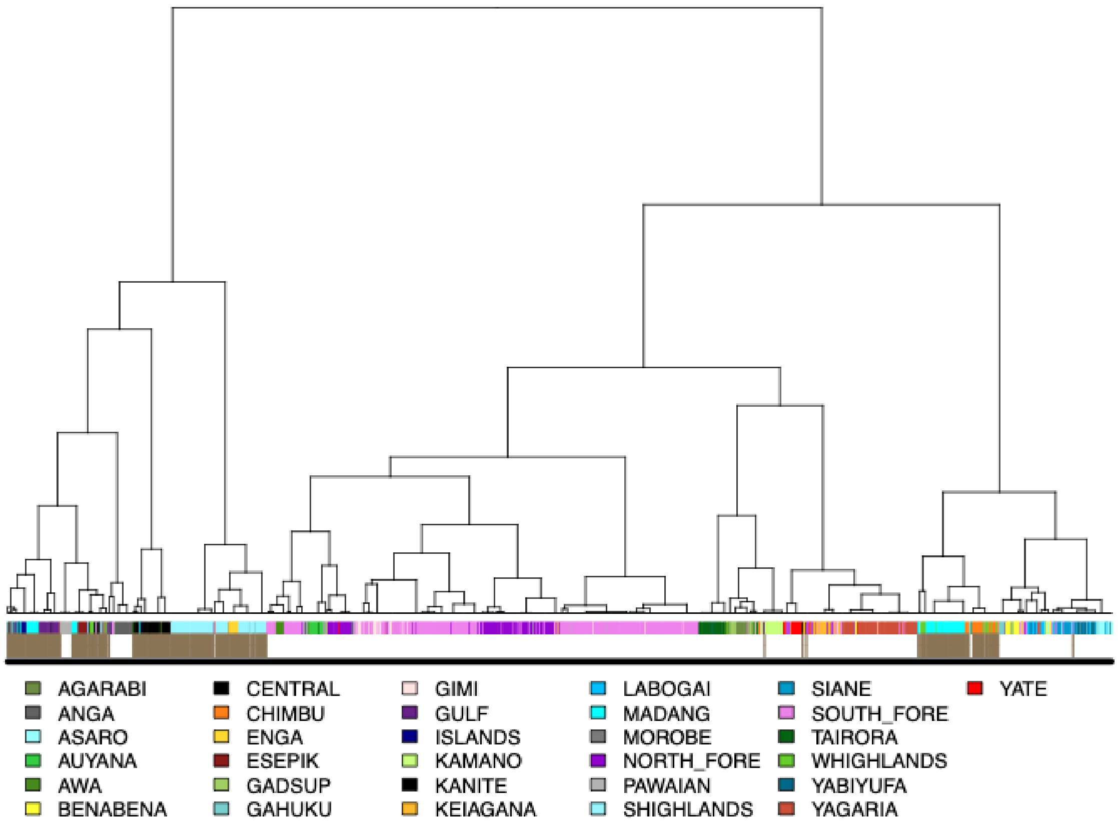

Figure S9 - FS Tree from CP analysis of 943 EPHNG individuals and 350 individuals from other PNG regions based on CP output (Figure S8). Light grey vertical lines connecting the horizontal black line to the linguistic group color labels highlight the 350 non EHPNG individuals. The majority of Individuals from Anga and Pawaian populations split from other EHPNG populations at K=2 of this tree with populations from other EHPNG regions. EHPNG Individuals from Siane, Gahuku, Asaro, Benabena and Yabiyufa linguistic groups (EHPNG Cluster 10 Figure S5, S6) in the north-west of the region split from remaining EHPNG populations at K=3 cluster with neighbouring non-EHPNG populations Chimbu and Madang.

POPULAITON	NUMBER OF SAMPLES	ORIGIN
EHPNG	1374	PRIMARY DATA
PNG	380	ACCESS AGREEMENT
ACB	95	1000 GENOMES
Altai	1	ANCIENT HOMININ
ASW	45	1000 GENOMES
Batwa	5	AFRICA
BEB	83	1000 GENOMES
CDX	82	1000 GENOMES
CEU	91	1000 GENOMES
CHB	103	1000 GENOMES
CHS	97	1000 GENOMES
CLM	93	1000 GENOMES
Colla	22	SOUTH AMERICA
Denisova	1	ANCIENT HOMININ
ESN	95	1000 GENOMES
FIN	99	1000 GENOMES
GB20	1	ANCIENT EUROPEAN
GBR	85	1000 GENOMES
GIH	96	1000 GENOMES
GWD	112	1000 GENOMES
IBS	107	1000 GENOMES
ITU	98	1000 GENOMES
JPT	104	1000 GENOMES
Khoesan_ColouredColesberg	20	AFRICA
Khoesan_ColouredWellington	20	AFRICA
Khoesan_GuiGhanaKgal	15	AFRICA
Khoesan_Juhoansi	18	AFRICA
Khoesan_Karretjie	20	AFRICA
Khoesan_Khomani	39	AFRICA
Khoesan_Khwe	17	AFRICA
Khoesan_Nama	20	AFRICA
Khoesan_SEBantu	20	AFRICA
Khoesan_SWBantu	12	AFRICA
Khoesan_Xun	19	AFRICA
KHV	98	1000 GENOMES

LBK	1	ANCIENT EUROPEAN
Loschbour	1	ANCIENT EUROPEAN
LWK	79	1000 GENOMES
MSL	69	1000 GENOMES
MXL	55	1000 GENOMES
PEL	76	1000 GENOMES
PJL	86	1000 GENOMES
PUR	104	1000 GENOMES
Pygmy_Baka_Cam	56	AFRICA
Pygmy_Baka_Gab	16	AFRICA
Pygmy_Bakiga	34	AFRICA
Pygmy_Batwa	27	AFRICA
Pygmy_Bongo_GabE	22	AFRICA
Pygmy_Bongo_GabS	24	AFRICA
Pygmy_Nzebi_Gab	20	AFRICA
Pygmy_Nzime_Cam	52	AFRICA
STU	96	1000 GENOMES
TSI	106	1000 GENOMES
Ust_Ishim	1	ANCIENT EUROPEAN
Wichi	19	SOUTH AMERICA
YRI	101	1000 GENOMES

Table S1 - Populations used in Phasing Panel.

Village breakdown of ‘Village dataset’

**LINGUISTIC GROUP** / VILLAGE	COUNT OF INDIVIDUALS
**AGARABI**	
KAINOA	17
**ANGA**	
BOIKO	3
DUNGWI	15
SIMBARI	2
**ASARO**	
GIMISEVI	18
**AUYANA**	
OIYANA	22
**AWA**	
TAUNA	11
**BENABENA**	
MATAUSA	24
**GADSUP**	
UKARUMPA	16
**GAHUKU**	
HORIPOKAVE	16
**GIMI**	
EMO	15
ETEVE	8
HAIYARU	1
UNKNOWN	4
**KAMANO**	
HOMORI	24
**KANITE**	
UNKNOWN	3
**KEIAGANA**	
HOGATERU	18
UWAMI	9
**LABOGAI**	
LOSAVE	19
**NORTHFORE**	
ANUMPA	76
AWANDE	21
KALU	16
OKAPA	1
UNKNOWN	11
**PAWAIAN**	
UNKNOWN	16
**SIANE**	

WAIFO	24
**SOUTHFORE**	
KETABI	1
AGAKAMATASA	12
AI	5
AMORA	3
AWAROSA	9
HIGITARU	1
ILESA	13
INTAMATASA	6
IVAKI	35
KALU	6
KAMATA	12
KAMIRA	16
KANIGITASA	23
KASARAI	3
KEIAKASA	2
KETABI	12
KUME	6
MENTILASA	9
MIARASA	9
MUGAIAMUTI	35
OMA-KASORU	3
PAITI	15
PUROSA-TAKAI	22
TAKAI	27
TAKARI	8
TAMOGAVISA	1
UMASA	6
UNKNOWN	23
URAI	4
WAISA	25
WANIKANTO	2
WANITABI	5
YAGAREBA	2
YAGUSA	1
YASUBI	4
**TAIRORA**	
BONTA	38
**YABIYUFA**	
HOIHATOBE	22
**YAGARIA**	
KIWURUGA	49
NUSAGUNA	38
**YATE**	

KAUNA	20
Grand Total	943

Table S2 - Table of village localities in the *Village Analysis* dataset of 943 individuals from 21 EHPNG linguistic groups.

Analysis	Figure	Original merge *	Number of sites	Filters	Number of individuals	Sites after filters
PCA	Figure 1b	Linguistic Group	699865	--geno 0.01 --remove Related inds --indep-pairwise 50 5 0.8 –maf 0.05	320	222812
ADMIXTURE	Figure S2	Linguistic Group	699865	--geno 0.01 --remove Related inds --indep-pairwise 50 5 0.8 –maf 0.05	320	222812
FST	Table S2	Linguistic Group	699865	--merge 1000 Genomes --geno 0.01 --remove Related inds	313	369464
ROH	Table S5, Figure S3	Linguistic Group	699865	--merge 1000 Genomes --geno 0.01 --remove Related inds –maf 0.05	313	365678
Proportion of heterozygous sites	Table S5, Figure S3	Linguistic Group	699865	--merge 1000 Genomes --geno 0.01 --remove Related inds –maf 0.05	313	365678
LD	Figure 2, Table S5	Linguistic Group	699865	--merge 1000 Genomes --geno 0.01 --remove Related inds --chr 22 --maf 0.05 (per population)	260	≈ 4,000 per population
CP/FS (1)	Figure 1a, Figure S4	Village Analysis	240774	--merge Phasing panel --geno 0.01 --remove Related inds	320	122663
CP/FS (2)	Figure 3, Figure S5, S6	Village Analysis	240774	--merge Phasing panel --geno 0.01 --remove Related inds	943	122663
CP/FS (3)	Figure S8, S9	Village Analysis	240774	--merge Phasing panel --geno 0.01 --remove Related inds	1293	122663
ADMIXTURE (2)	Figure S7	Village Analysis	240774	--merge 1000 Genomes --geno 0.01 --remove Related inds --indep-pairwise 50 5 0.4	943	77828
SOURCEFIND	Table S6 S7	Village Analysis	240774	--merge Phasing panel --geno 0.01 --remove Related inds	1293	122663

Table S3 - Table showing sites filters for each analysis.

Table S4 - Has been provided as a separate table.

F_{ST} analysis of 20 EHPNG linguistic groups after 7 outlier individuals identified in PCA were removed.

Population	Mean proportion of heterozygous sites	Mean total ROH (> 1Mb)	Average LD	Mean pairwise relatedness
Anga	0.336	226	0.167	0.039
Pawaian	0.342	179	0.164	0.073
Tairora	0.341	208	0.175	0.055
Auyana	0.345	172	0.162	0.064
Awa	0.343	186	0.173	0.056
Gadsup	0.351	143	0.152	0.057
Agarabi	0.349	156	0.173	0.057
Kamano	0.350	150	0.154	0.054
southFore	0.351	146	0.152	0.044
Gimi	0.353	126	0.149	0.05
northFore	0.355	123	0.155	0.058
Keiagana	0.354	129	0.148	0.055
Yate	0.352	133	0.144	0.049
Yagaria	0.353	131	0.151	0.06
BenaBena	0.352	130	0.145	0.038
Labogai	0.356	107	0.145	0.057
Yabiyufa	0.354	110	0.14	0.053
Siane	0.355	110	0.146	0.059
Gahuku	0.355	109	0.145	0.052
Asaro	0.354	118	0.147	0.051

Table S5 - Summary of genetic statistics for 20 EHPNG linguistic groups. Groups are ordered according to geography in the same way as in the F_{ST} table (Figure S4). Runs of Homozygosity (ROH) is the mean total length of long runs of homozygosity (>1Mb) per individual in each population.

	EHPNG FS Cluster *Village Analysis* dataset											
**LINGUISTIC GROUP**	**1**	**2**	**3**	**4**	**5**	**6**	**7**	**8**	**9**	**10**	**11**	**12**
**ANGA**	0	0	0	0	0	0	0	0	22	0	0	0
**PAWAIAN**	0	0	0	0	2	13	0	1	0	0	0	0
**TAIRORA**	1	0	2	0	0	0	0	0	1	1	0	33
**AUYANA**	21	0	0	0	0	0	0	0	0	0	0	1
**AWA**	1	0	0	0	0	0	0	0	0	0	10	0
**GADSUP**	2	0	13	0	0	0	1	0	0	0	0	0
**AGARABI**	0	0	17	0	0	0	0	0	0	0	0	0
**KAMANO**	0	0	24	0	0	0	0	0	0	0	0	0
**SOUTH FORE**	3	3	4	112	178	1	18	14	2	7	20	0
**GIMI**	0	0	0	20	6	0	1	0	1	0	0	0
**NORTH FORE**	27	2	0	8	1	1	10	76	1	0	0	0
**KANITE**	0	0	1	0	0	0	2	0	0	0	0	0
**KEIAGANA**	0	0	0	0	0	0	26	2	0	0	0	0
**YATE**	1	0	1	0	0	0	18	0	0	0	0	0
**YAGARIA**	0	1	0	1	0	1	84	0	0	0	0	0
**BENABENA**	0	18	1	0	0	0	4	0	0	0	0	1
**LABOGAI**	0	16	0	0	0	0	0	0	0	3	0	0
**YABIYUFA**	0	6	0	0	0	0	0	0	0	15	0	1
**SIANE**	0	1	0	0	0	0	0	0	0	23	0	0
**GAHUKU**	0	5	0	0	0	1	0	0	0	10	0	0
**ASARO**	0	2	0	0	0	0	0	0	0	16	0	0

Table S6 – Breakdown of FS clustering in *Village Analysis* dataset (n=943); K=12.

Table S7 is provided as a separate excel sheet
