# Supplementary material for: Population structure and migration in the Eastern Highlands of Papua New Guinea, a region impacted by the kuru epidemic
Source: Am J Hum Genet. 2024 Mar 19;111(4):668–79. doi: 10.1016/j.ajhg.2024.02.011 (PMC11023820; doi:10.1016/j.ajhg.2024.02.011)
Supplement: Document S2. Article plus supplemental information [file mmc3.pdf]

Population structure and migration in the Eastern Highlands of Papua New Guinea, a region impacted by the kuru epidemic

Authors

Liam Quinn, Jerome Whitfield, Michael P. Alpers, ..., John Collinge, Garrett Hellenthal, Simon Mead

Correspondence

j.collinge@prion.ucl.ac.uk

Explore an in-depth investigation of the Eastern Highlands of Papua New Guinea. Analyses attempt to disentangle the complex relationships between genetics, linguistics, and the clan system of political organization. Additionally, an analysis investigating the impact of the kuru epidemic on migration flows in the region is undertaken.

Quinn et al., 2024, The American Journal of Human Genetics *111*, 668–679
April 4, 2024

© 2024 The Authors.

https://doi.org/10.1016/j.ajhg.2024.02.011

ARTICLE

Population structure and migration in the Eastern Highlands of Papua New Guinea, a region impacted by the kuru epidemic

Liam Quinn,[1,2] Jerome Whitfield,[1] Michael P. Alpers,[3,4] Tracy Campbell,[1] Holger Hummerich,[1] William Pomat,[4] Peter Siba,[4] George Koki,[4] Ida Moltke,[2,6] John Collinge,[1,6,*] Garrett Hellenthal,[5,6] and Simon Mead[1,6]

Summary

Populations of the Eastern Highlands of Papua New Guinea (EHPNG, area 11,157 km^2) lived in relative isolation from the rest of the world until the mid-20th century, and the region contains a wealth of linguistic and cultural diversity. Notably, several populations of EHPNG were devastated by an epidemic prion disease, kuru, which at its peak in the mid-twentieth century led to some villages being almost depleted of adult women. Until now, population genetic analyses to learn about genetic diversity, migration, admixture, and the impact of the kuru epidemic have been restricted to a small number of variants or samples. Here, we present a population genetic analysis of the region based on genome-wide genotype data of 943 individuals from 21 linguistic groups and 68 villages in EHPNG, including 34 villages in the South Fore linguistic group, the group most affected by kuru. We find a striking degree of genetic population structure in the relatively small region (average F_{ST} between linguistic groups 0.024). The genetic population structure correlates well with linguistic grouping, with some noticeable exceptions that reflect the clan system of community organization that has historically existed in EHPNG. We also detect the presence of migrant individuals within the EHPNG region and observe a significant excess of females among migrants compared to among non-migrants in areas of high kuru exposure ($p = 0.0145$, chi-squared test). This likely reflects the continued practice of patrilocality despite documented fears and strains placed on communities as a result of kuru and its associated skew in female incidence.

Introduction

The Eastern Highlands region of Papua New Guinea (EHPNG) that covers an area of 11,157 km^2 was more or less isolated from the rest of the world until the early decades of the 20th century.[1] At the beginning of the 20th century, western observers held the belief that the highlands of New Guinea were largely uninhabited.[2] Exploration by Christian missionaries and gold prospectors, however, revealed the highlands to contain heavily populated valleys that were home to close to one million people.[3,4] From 1918 until the independence of Papua New Guinea in 1975, the region was subjected to Australian colonial rule.[5] Colonial authorities divided the peoples of EHPNG into administrative groups based on the language spoken in particular areas and to date, most studies of the people in EHPNG and the PNG Highlands in general have used these linguistic groupings as convenient population labels.[1]

The people of EHPNG are notable for their complex cultural[6,7] and trade systems,[8] cosmology, and linguistic diversity.[1,9] The number of separate languages spoken in the broader highlands of PNG is believed to be in the hundreds and in the EHPNG region alone there are 37 distinct linguistic groups.[1] Historically, each linguistic group consisted of clans,[10,11] with clan composition dynamic, depending on clan disputes and other cultural factors.[12] The region has extreme terrain including mountains, valleys, and fast-flowing river systems that impeded travel.[1,13] Yet, anthropological studies have pointed to a complex picture of migration of individuals and groups over both long and short distances within EHPNG.[12,14] In addition to this, theories abound from other fields of study about possible admixture within wider Melanesia and Australia: linguists, archaeologists, and historians have discussed competing theories as to how these regions were originally populated and the origin of the current groupings.[15–17] The presence of non-indigenous products in EHPNG evidence long-distance trade networks, proving EHPNG not to have been completely isolated, and that trade may have led to genetic exchange.[1] Furthermore, there are hypotheses of migration both within and into the region in the literature.[18,19] Genetic data could help reveal how factors like linguistic diversity, cultural and agricultural practices, migration, and extreme geography can shape population genetic structure.

Several of the linguistic groups in the EHPNG (mainly the Fore and groups with whom the Fore traditionally

[1]MRC Prion Unit at UCL, Institute of Prion Diseases, UCL, London, UK; [2]The Bioinformatics Centre, Department of Biology, University of Copenhagen, Copenhagen, Denmark; [3]Health Sciences, Curtin University, GPO Box U1987, Perth, WA 6845, Australia; [4]Papua New Guinea Institute of Medical Research, PO Box 60, Goroka, Eastern Highlands Province, Papua New Guinea; [5]University College London Genetics Institute, Darwin Building, Gower Street, London WC1E 6BT, UK
[6]Senior author
*Correspondence: j.collinge@prion.ucl.ac.uk
https://doi.org/10.1016/j.ajhg.2024.02.011.
© 2024 The Authors. This is an open access article under the CC BY license (http://creativecommons.org/licenses/by/4.0/).

intermarried) were devastated by an epidemic of the prion disease kuru during the 20th century.[4,20] Prion diseases are fatal transmissible neurodegenerative disorders caused by the propagation of prions, infectious agents composed of assemblies of misfolded prion protein.[21] Kuru was transmitted by endocannibalistic mortuary feasts at which a deceased relative was consumed by kith and kin in a ritualistic manner.[6] The epidemic resulted in 2,700 recorded deaths and predominantly affected adult women and children of both sexes because they consumed the most highly prion-infected tissues.[4] During the height of the epidemic, observers noted many villages were depleted of adult women.[22] Hence the epidemic likely had an impact on established cultural and demographic processes, like migration in the region.[23] Moreover, kuru imposed strong selection pressure on the affected populations, with evidence of strong balancing selection acting at the prion protein gene locus.[24] Whether kuru imposed selection pressure at other loci is currently unknown, but a prerequisite of answering this question is a deeper understanding of the population structure of the region.

To date, relatively few genetic studies of the EHPNG region have been performed.[19,25,26] These have shown that groups that populate the broader highlands region of PNG display high levels of genetic differentiation.[18,19] However, no studies have yet had enough data from EHPNG to fully explore population diversity, structure, and migration in this subregion of the highlands, and its correlations with language, topography, and kuru disease. Motivated by this, we analyzed genetic data for 943 individuals from EHPNG sampled from 21 of the 37 distinct linguistic groups in the region. Moreover, with dense sampling from a total of 68 villages, we have fine-scale village-level data for several of the linguistic groups, including the kuru-affected Fore. We present an in-depth analysis of the population structure in EHPNG that reveals a striking complexity of the small region. Subsequently, we apply knowledge of this population structure to address questions regarding the population genetic impact of kuru. Finally, we discuss the likely causes of this complex population structure, what it can tell us about the history and population dynamics of the region, and what this might entail for potential future studies of EHPNG and of kuru.

Material and methods

Ethical aspects

Laboratory studies were approved by the Papua New Guinea Medical Research Advisory Committee and by the local Research Ethics Committee of UCL Institute of Neurology. Participation of the communities involved was established and maintained through discussions with village leaders, communities, families, and individuals. The field studies followed the principles and practice of the Papua New Guinea Institute of Medical Research (PNGIMR), which included individual oral consent from all participants before any samples were obtained.

EHPNG samples and genome-wide genotyping

Blood samples were taken from 4,217 individuals from communities in EHPNG by members of PNGIMR. Information was obtained about the individual's village of residence, language spoken in that village, and the sex of the individual. After initial processing locally, these samples were transported to the Medical Research Council Prion Unit (MRCPU) in the United Kingdom. The samples were then further processed and genotyped in several stages. The first 488 samples were genotyped on the Illumina 670 genotyping platform. The following 1,106 samples were genotyped on the Illumina Omni Express genotyping platform. 83 individuals were genotyped on both the Illumina 670 (678,000 variants) and Illumina Omni Express (748,000 variants) platforms to permit downstream analysis of possible biases introduced by the different genotyping platforms, leaving 1,511 different individuals in the primary dataset.

Other samples and genotype data used for context

In addition to the EHPNG samples, we also included samples from previous studies in a subset of the analyses. Specifically, we included 380 samples[19] from 84 PNG linguistic groups and surrounding islands. Among these samples were 26 individuals from 10 EHPNG linguistic groups. All of these 380 samples were genotyped using the Illumina Infinium Multi-Ethnic Global Array (MEGA). We also included samples from the IBS (Spanish), FIN (Finnish), CEU (Americans of European descent), CHB (Han Chinese), and YRI (Yoruba Nigerian) populations from the 1000 Genomes Project. For these populations we downloaded phase 3 Omni genotyped data from the International Genome Sample Resource portal (https://www.internationalgenome.org/category/omni/).[27] Additional data were added to create a phasing panel with as much population breadth as possible. In addition to the EHPNG data, 1000 Genomes phase 3 data (all samples in addition to the five populations above), data obtained through separate group access agreements, including 33 African populations, 2 indigenous South American populations, 4 ancient European individuals, a Denisovan, and a Neanderthal individual, were merged. This gave a haplotype phasing panel of 4,632 individuals (see Table S1 for breakdown).

Relatedness checks of primary data

Relatedness checks were performed on the primary dataset of 1,511 individuals from 21 linguistic groups in EHPNG. A relatedness threshold was applied in PLINK 1.9[28] of PIHAT <0.2 (using the –genome option after data for each linguistic group were linkage disequilibrium pruned with the command –indep-pair-wise 50 5 0.4 to extract between 70,000 and 75,000 independent markers for relatedness analysis). After using this threshold, 943 individuals from 21 linguistic groups remained for further analysis (see Table S2 for breakdown of samples by locality). For samples used from other sources, only unrelated samples were used based on the relatedness analysis thresholds used in the publications they were based upon.

Datasets used for analysis and nomenclature

Analyses were performed on two datasets based on the primary collection of EHPNG data after the removal of related individuals. These are subsequently referred to as the "linguistic group analysis" dataset and the "village analysis" dataset. The linguistic group analysis dataset concerns analyses performed on genome-wide genotype data, free from the effects of genotype platform

batch bias and unequal sample size effects. This was achieved by taking 16 individuals from the 20 linguistic groups that had more than three samples, who were all genotyped on the same platform, leaving 320 individuals. The village analysis dataset uses the whole dataset of 943 individuals merged across multiple genotyping platforms and is based primarily on methods that require phased genotype data. It contains 623 additional individuals, predominantly from the kuru-affected region (see Figure 1A for map of region). We analyzed these data using haplotype-based approaches, which can achieve finer resolution[29] and alleviate concerns of ascertainment bias that arise from using chip data.[30]

Below, more details are provided for each of the analyses performed using each of the two datasets. For a detailed summary of sites, samples, and filters used for each analysis in this investigation, please refer to Table S3.

Principal components analysis

We performed principal components analysis (PCA) using the PLINK 1.9 command '—pca', on the linguistic group analysis dataset. PCA revealed numerous individuals who clustered distantly from others in their own linguistic groups. To avoid any potential effect on F_{ST}, LD, ROH, heterozygosity, ADMIXTURE, and relatedness analyses of individuals who have likely moved across the region since European contact, we removed the seven most extreme of the outliers from these analyses (circled in Figure 1B), leaving 313 individuals. We identified these outliers as individuals with a value that is 3.2 × IQR greater than the third quartile or 3.2 × IQR less than the first quartile on either PC1 or PC2 within each linguistic group. We on purpose used stricter outlier detection criteria than usual in an attempt to avoid biasing the results by accidently removing natural variation within each linguistic group, which was not caused by individuals who have moved across the region since European contact.

Tree of relationship of EHNPG languages

An unrooted bifurcating tree of 20 EHPNG languages representing the linguistic groups in our analyses was created. This was done using the glottolog tool and is based on consensus estimates based on accepted classifications of the languages.

Pairwise F_{ST} estimations of EHPNG linguistic groups

Pairwise F_{ST} calculations were performed between linguistic groups of the remaining 313 individuals from the 20 linguistic groups in the linguistic group analysis dataset. This was done in PLINK 1.9 using the –fst command after applying a missingness filter using the –geno command. As a comparison, 13 random individuals were taken from the 1000 Genomes populations IBS (Iberian Spanish) and FIN (Finnish) using the same SNPs as in our analysis.

Linkage disequilibrium decay curves

For linkage disequilibrium (LD) decay curves, all linguistic groups in the linguistic group analysis dataset were downsampled to 13 individuals to give equal numbers of individuals per linguistic group (after taking into account the observed outlier individuals in PCA), as these statistics are impacted by sampling size differences. LD decay curves were created for each of the linguistic groups. This was performed using data from chromosome 22 and the commands (–ld-window 1000000, –ld-window-kb 600, –ld-window-r2 0, –maf 0.05, –r2 dprime with-freqs). The same analyses were also performed on 13 randomly chosen individuals from each of the 1000 Genomes populations CEU (Northern Europeans from Utah) and YRI (Yoruba Nigeria) to allow comparison to other populations outside PNG. Average LD over 300 kb was calculated to allow comparisons between populations.

Runs of homozygosity and proportion of heterozygous sites

Proportion of heterozygous sites per individual in the linguistic group analysis dataset was calculated using the –het command in PLINK 1.9 (excluding the seven outlier individuals highlighted in Figure 1B). Total runs of homozygosity (ROH) length values for each individual in the linguistic group analysis dataset (excluding the seven outliers identified in Figure 1B) were also calculated using PLINK 1.9 and the –homozyg setting. A sliding window of 50 SNPs was used and a SNP density of 50 SNPs per kilobase, and a minimum ROH length threshold of 1 Mb was applied. Mean total length in Mb per linguistic group were then tabulated.

ADMIXTURE

We ran ADMIXTURE on the linguistic group analysis dataset. We ran the analysis up to 500 times in order to check for statistical convergence. Statistical convergence was achieved for $K = 2$ to $K = 13$ but not for higher K values. The convergence criterion was defined as observing 3 runs with the differences of maximum likelihood less than 5 units. If there were 3 runs with log likelihood differences less than 5, the process stopped before reaching 500 iterations.

We tested for admixture between all 943 individuals in the EHPNG village analysis dataset and 1000 Genomes CEU (94 individuals) and CHB (103 Han Chinese from Beijing individuals) populations using ADMIXTURE[31] with $K = 3$. The analyses of each dataset were run with the same convergence criteria as the ADMIXTURE analysis performed on the linguistic group analysis dataset (see above).

Phasing of the EHPNG data

Our phasing panel consisting of 4,632 individuals, including 1,374 from EHPNG, and contained 122,662 variants available for phasing after a missingness filter of –geno 0.01 was applied in PLINK 1.9 (see Table S1 for populations used in phasing panel). We then jointly phased the autosomal chromosomes for all individuals using SHAPEIT[32] with default parameters and the linkage disequilibrium-based genetic map build 37.

ChromoPainter and fineStructure analyses

Three ChromoPainter and complementary fineStructure analyses were performed using different subsets of the village analysis dataset.[29] The first was performed using the same 320 individuals as from the linguistic group analysis dataset to provide a complementary analysis based on phased data methods. A second ChromoPainter/fineStructure analysis was performed using all 943 EHPNG samples available to explore population structure at a fine-scale in the region, taking advantage of the dense sampling of villages particularly in the kuru-affected region. A third analysis included an additional 350 individuals from other PNG regions to examine the contribution of these groups to population structure in EHPNG.

ChromoPainter is a "painting" technique that compares haplotype patterns within a target chromosome to those within a set of sampled "donor" chromosomes. In a genetic region, if a target's haplotype patterns are more similar to a particular donor relative

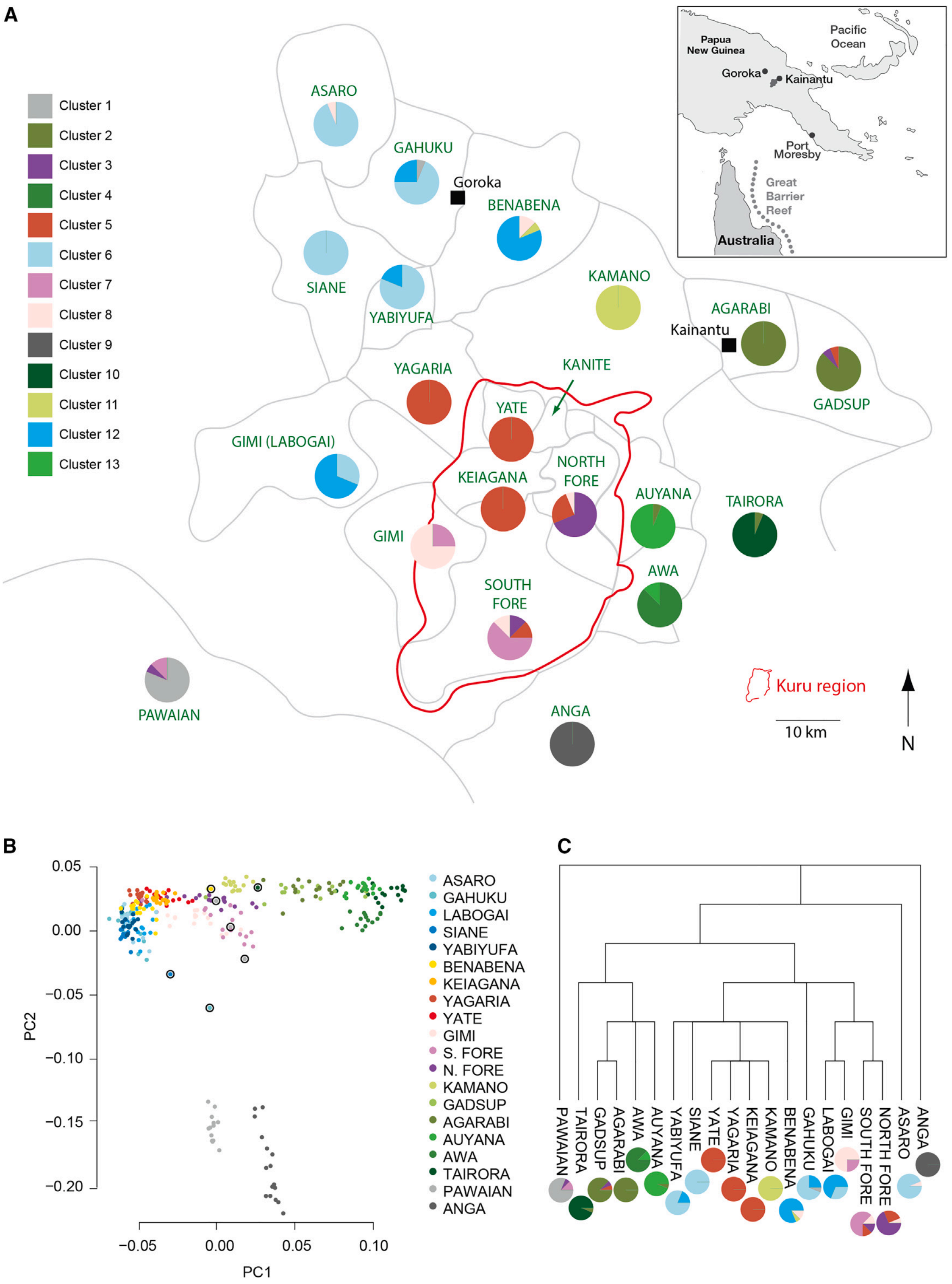

Figure 1. Population structure of EHPNG described through linguistic group membership

(A) Map of 20 sampled linguistic groups in EHPNG region, with pies indicating the proportion of individuals in each region assigned to each fineStructure cluster (colors). The Kanite, the 21st linguistic group in the village analysis dataset, are also shown on the map but were excluded from population structure analysis as n = 3. Arrows indicate individuals highlighted in PCA analysis who are positioned far away from other individuals in the same linguistic group.

(B) PC1 and PC2 from a PCA analysis of the same 320 individuals as in the fineStructure analysis in (A). Circled individuals are placed distantly from others from the same linguistic group.

(C) Best estimate language tree for 20 EHPNG linguistic groups (source http://glottolog.org). fineStructure clustering profiles for each linguistic group are placed alongside their labels.

to the other donors, this suggests the target shares a more recent ancestor with that donor relative to the others for that genetic region. ChromoPainter provides a "painting profile" for each target individual reflecting the amount of genome-wide DNA for which the individual is inferred to share a most recent ancestor with each donor individual.

For each analysis, we painted each of the individuals as a recipient when using all other individuals in the same dataset as donors. To do so, we first estimated two ChromoPainter model parameters, the switch ("-n") and emission ("-M") rates, by painting each recipient individual for chromosomes 1, 5, 7, 15, and 21 while using 10 Expectation-Maximization (E-M) iterations ("-i 10 -in -iM"). Within each individual, we averaged the estimated values of the switch and emission rates across these five chromosomes, weighting by SNP number. We then averaged these values across all individuals and re-painted each recipient individual while fixing these average switch and emission values, giving our final painting of each person. The fixed {switch, emission} values used for these final ChromoPainter runs were {50.230, 0.000169} for the linguistic group analysis, {26.076, 0.000103} for the village analysis dataset, and {55.214, 0.000328} for the village analysis dataset + external samples. For the linguistic group analysis and village analysis dataset, we ran ChromoPainter with "-k 15" to use when calculating the normalization parameter "c" in fineStructure, while we ran ChromoPainter with "-k 50" for the village analysis dataset and external samples.

We next used fineStructure[29] to group individuals into genetically homogeneous clusters based on the ChromoPainter output of the three datasets. Importantly, these groupings are free from any bias due to *a priori* classifications of individuals, e.g., based on linguistic group classifications. Following the recommended fineStructure approach described by a previous publication,[29] we inferred a normalization parameter c and performed two million iterations of Markov Chain Monte Carlo (MCMC), sampling an inferred clustering every 10,000 iterations after a burn-in of one million iterations. Starting from the single MCMC sampled clustering with highest posterior probability, we then performed 100,000 additional hill-climbing steps in fineStructure to find a nearby state with even higher posterior probability. fineStructure then created a bifurcating tree that presents the relationship of these clusters to one another, using a greedy approach that merged two clusters at a time based on which merging minimized the decrease in posterior probability.

SOURCEFIND

We used SOURCEFIND to form the painting profile of each of the 12 EHPNG clusters identified in the village analysis dataset painting (Figures S5 and S6) as a mixture of other groups.[33] SOURCEFIND uses a Bayesian approach that puts a prior on the number of groups contributing > 0% to this mixture, hence eliminating contributions that cannot be reliably distinguished from background noise. For this analysis, we used the painting profiles from the external analysis painting (Figure S8). Specifically, we used SOURCEFIND to form each EHPNG cluster's painting profile as a mixture of those of 21 ancestry surrogate groups: the 10 non-EHPNG geographically labelled groups (as defined in a previous study) and the 11 other EHPNG clusters. The painting profile of each population was defined as a vector with 25 values, corresponding to the average amount of DNA that members from that population match (as inferred by ChromoPainter) to all individuals in the 12 EHPNG clusters, the 10 non-EHPNG groups, and each of the single Anga, Gahuku, and Tairora individuals from Bergström et al.[19] included in this painting. We ran SOURCEFINDv2 with default settings, which ran for 200,000 MCMC iterations, sampling the posterior values of surrogates' contributions every 5,000 iterations after 50,000 iterations discarded as "burn-in." A maximum of 8 surrogate groups were allowed to contribute at any iteration, with an expected value of 4 surrogates contributing and surrogates' contributions rounded to the nearest whole percent. Our final results reported average contributions across the posterior samples.

Testing for sex-biased migration in the kuru-affected region

To test whether there was a sex-bias among migrants into kuru-affected areas of EHPNG, we identified migrants in our fineStructure analysis of the village analysis dataset comprising 943 EHPNG individuals from 21 linguistic groups. We did not use PCA to highlight migrants in this dataset due to the extensive sample size biases in the dataset which can greatly impact PCA. Migrants were classified using fineStructure clustering K = 12 (Figure S6; Table S6) at the village level. This level of clustering was selected as it allowed the identification of individuals originating from distinct populations to where they were sampled and not due to very local population structure. In each village a "migrant" was defined as someone who was in a genetic cluster at K = 12 that did not match the cluster assignment of the majority of the other individuals in that village. We excluded villages that had fewer than four individuals and individuals where village information was not available. One caveat to this was based on the formation of two clusters in the village analysis dataset based on two South Fore dialect groups (clusters 4 and 5). This resolution was only possible due to the bias of sampling within the South Fore linguistic area, so individuals would not be classed as migrants if they were placed in cluster 4 while residing in a predominantly cluster 5 village, and vice versa. After filtering for low-sample-size villages and individuals lacking sex or village information, 866 individuals remained. We then divided the dataset into three cohorts of kuru exposure based on kuru incidence in village localities in EHPNG: high (276 individuals from 19 villages), medium (201 individuals from 12 villages), and low/zero (389 individuals from 17 villages). These cohorts were based on previously published exposure indices from a 2009 study of kuru that assessed colocalization of genetic variants with kuru incidence.[24] In each of the three kuru disease cohorts, we then used a chi-squared statistic to test whether there were more female migrants than expected given the proportion of females among the sampled non-migrants.

To test whether the effect of sex on the chance of being a migrant significantly differed between the three kuru exposure groups, we compared the following logistic regression models by performing a likelihood ratio test with 2 degrees of freedom using the anova function in R:

$$\log(yi / (1\text{-}yi)) = \beta\text{intercept} + \beta\text{sex sexi} + \beta\text{kuru kurui}$$

$$\log(yi / (1\text{-}yi)) = \beta\text{intercept} + \beta\text{sex sexi} + \beta\text{kuru kurui} + \beta\text{sex} \times \text{kuru sexi} \times \text{kurui}$$

where yi is the probability that individual i is a migrant, sex_i is the sex of individual i, and $kuru_i$ is the level of kuru incidence in the

area where individual i was sampled (no/low, medium, or high, modeled as an ordinal variable and with betas as effect sizes).

Results

Analyses of population structure of EHPNG region

To infer large-scale population structure in EHPNG (Figure 1) while controlling for effects of sampling size,[34] we analyzed a reduced dataset of 320 individuals containing 16 individuals from each of 20 linguistic groups, which we refer to as the linguistic group analysis dataset (see material and methods).

First, we performed principal components analysis (PCA) and plotted the results with individuals colored according to their linguistic group membership (Figure 1B). Principal component (PC) 1 and PC2 revealed that individuals from the same linguistic group broadly clustered together. PC1 correlated with placement of linguistic groups along a northwest-southeast cline, whereas PC2 separated the Anga and Pawaian linguistic groups from the other groups. Notably, the linguistic groups that overlapped on the plot tended to be geographically neighboring linguistic groups, for example individuals from linguistic groups in the northwest of the region (Asaro, Siane, Yabiyufa, Labogai, and Gahuku) clustered closely together. Examination of further PCs showed that several of the other linguistic groups separated out on some of the higher PC axes, including Tairora, Awa, Auyana, Kamano, Agarabi, and Gadsup, all groups situated in the eastern part of the region (see Figure S1 for PC3–10). Other linguistic groups, including the groups in the northwest, did not entirely separate out within the first 10 PCs. It is also worth noting that in the PC1-PC2 plot there were several clear outlier individuals including three Pawaian, one Benabena, one Gahuku, one Siane, and one Tairora individual who did not cluster near other individuals from the same linguistic group and may represent migrant individuals or descendants of migrants within the region who moved across the region after European contact (Figure 1B).

Next we estimated genetic differences between groups using pairwise F_{ST} values from the linguistic group analysis dataset after removing the above-mentioned seven outliers (Figure 1B; Table S4). On average, the pairs of linguistic groups had greater genetic differences than those found between groups of individuals from nations in Europe as distant as Finland and Spain (average F_{ST} in EHPNG 0.024 vs. 0.011 for Finland and Spain [>3,000 km]) when estimated using the same sites, despite being on average only 45 km apart. Consistent with the PCA analysis results, the Anga and Pawaian groups had the largest average pairwise F_{ST} values to other groups (Anga average 0.046, range 0.039–0.060; Pawaian average 0.050, range 0.043–0.064). At the other end of the spectrum, smaller-than-average values were seen between the linguistic groups in the northwest of the region that grouped together in the PC1-PC2 plot (average 0.0037, range 0.0021–0.0057). Additionally, we observed very small values for a few of the geographically neighboring group pairs like the South Fore and Gimi. More broadly, the affinities and differences between groups according to F_{ST}, combined with the PCA results, seemed to suggest genetic structure that roughly follows linguistic grouping (Figure 1C for linguistic tree of 20 languages) as well as the geographical sub-regions in EHPNG: northwest (blue colors in PCA), upper-mid (red colors in PCA), lower-mid (purple colors in PCA), northeast (light green colors in PCA), southeast (dark green colors in PCA), and south (gray colors in PCA), Pawaian, and Anga.

Next, to further assess the genetic structure among the linguistic groups and the presence of potential migrants, we ran ADMIXTURE on the linguistic group analysis dataset assuming the number of ancestral populations (K) is equal to 2–20. Statistical convergence was reached for K = 2–13. Notably, the clustering for K = 13 (Figure S2) showed a similar pattern to the F_{ST} and PCA results with greater heterogeneity and cleaner clustering of linguistic groups in the southeast compared to the northeast. Additionally, all the individuals highlighted as clear outliers and thus possible migrants in PCA displayed ADMIXTURE profiles that differed from the profiles of the other samples from the same linguistic groups.

As haplotype-based approaches can sometimes capture more fine-scale structure than techniques like PCA and ADMIXTURE,[29] we next phased data using SHAPEIT and then used ChromoPainter to infer the genome-wide proportion of haplotype segments for which each of 320 EHPNG individuals shares a most recent ancestor with each of the other 319 individuals. Visual inspection of these proportions (Figure S4) confirmed the structure inferred by PCA, ADMIXTURE, and F_{ST}, with increasing heterogeneity between linguistic groups along the northwest to southeast cline.

We next used fineStructure to assign individuals into discrete clusters based on the patterns of recent ancestry sharing inferred by ChromoPainter. While the maximum posterior sample from fineStructure assigned the 320 individuals into 38 clusters, fineStructure then merged these clusters sequentially to generate a bifurcating tree. At the level of the fineStructure tree with 13 groups, individuals fell into groups broadly consistent with the patterns observed in F_{ST} and PCA and ADMIXTURE (Figure 1C). Moreover, several individuals clustered outside of their own linguistic group at this level of the tree, in a manner broadly consistent with the clear outliers observed in PCA and supporting evidence of recent migration within the region.

To further investigate genetic differences between linguistic groups, we measured the mean proportion of heterozygous sites, mean length of runs of homozygosity (ROH) above 1 Mb, and linkage disequilibrium (LD) within each of the 20 EHPNG linguistic groups in the linguistic group analysis dataset (Table S5). As was done for F_{ST} calculations, we excluded the most extreme outliers identified

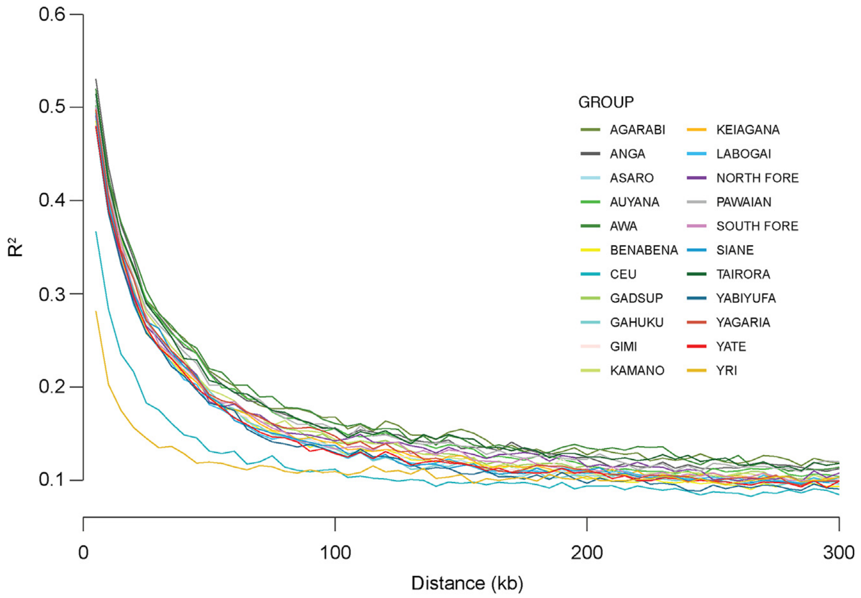

Figure 2. LD decay curves for 20 EHPNG linguistic groups
13 individuals analyzed per linguistic group, and 13 individuals from each of the 1000 Genomes YRI and CEU populations whose curves are below the EHPNG curves.

using PCA to avoid potential recent migrants affecting results, and for LD we subsequently downsampled all groups to 13 individuals to obtain the same number of samples from each linguistic group. For the proportion of heterozygous sites per linguistic group, we observed a trend of decreasing values when moving through the region from northwest to southeast and south (Figure S3A) with especially the groups in the southeast and south being lower than the rest. The fraction of ROH followed the same trend, but with an increase instead of a decrease and thus with the groups in southeast and east being the highest (Figure S3B). The latter did not seem to be driven by differences in relatedness (Figure S3C). Consistent with the trends observed for the proportion of heterozygous sites and fraction of ROH, groups at the southern fringes, east, and southeast exhibit higher LD (Table S5; Figure S3D). Notably, LD seemed to decay over physical distance much slower in the EHPNG groups than in CEU or YRI samples (Figure 2), likely reflecting the region's history of isolation and small population sizes.

Analysis of population structure at "FineScale"

The above analyses using the linguistic group analysis dataset permitted investigation of the relevance of the linguistic group classification in defining population structure without concerns of sample size biases impacting inferences. However, our data collection has an additional 623 samples largely from multiple villages within the North and South Fore groups, which allowed us to explore population structure at a finer scale. This village analysis dataset contained 940 individuals from the same 20 EHPNG linguistic groups plus 3 individuals from a 21st linguistic group, the Kanite.

We used ChromoPainter/fineStructure to cluster these 943 EHPNG individuals, with the resulting groupings largely mirroring the linguistic group analysis (Figures S5 and S6). However, within several groups we observed notable population differentiation at the village level (Figure 3). For example, when considering the level of the fineStructure tree where there are only four groups, one such group predominantly contained South Fore individuals from villages speaking the Pamusagina dialect and another predominantly contained North and South Fore individuals from villages speaking Atigina and Ibusa dialects. When considering the level of the fineStructure tree where there are 29 groups, individuals from the South Fore village of Ilesa clustered more with people from a nearby Awa village than with people from other South Fore villages. Similarly, at this level of clustering individuals in the village Kalu in the North Fore appeared more genetically related to the Auyana than to people from the other two North Fore villages. Furthermore, people from the two sampled Yagaria villages largely clustered separately, and people from the two Anga sampled villages clustered completely separately.

Relationship of EHPNG populations to other groups

We next investigated the relationship of EHPNG individuals and groups to external populations in order to assess whether migration from and admixture with such populations has played a role in shaping the population structure in the EHPNG described above and also to allow contextualization of evidence of migration on a broader scale.

As EHPNG groups came into contact with Europeans in the 20th century for the first time and contact with Europeans has led to admixture in many other places in the world, we included Europeans among potential external populations from outside PNG. We also included East Asians due to their geographical proximity and to serve as a proxy for Austronesian ancestry. Specifically, we performed an ADMIXTURE analysis of all 943 individuals in the village analysis dataset combined with all the 1000 Genomes individuals from CEU (north Americans with European ancestry) and CHB (Chinese individuals from Beijing) assuming the presence of 3 ancestral populations (K = 3). This analysis (Figure S7) revealed minor amounts of ancestry related to the two external sources in almost all EHPNG individuals and this amount was similar within each group, which is inconsistent with recent admixture. Only four individuals showed elevated levels (10%–20% admixture proportions) from the external sources, represented mainly by CHB.

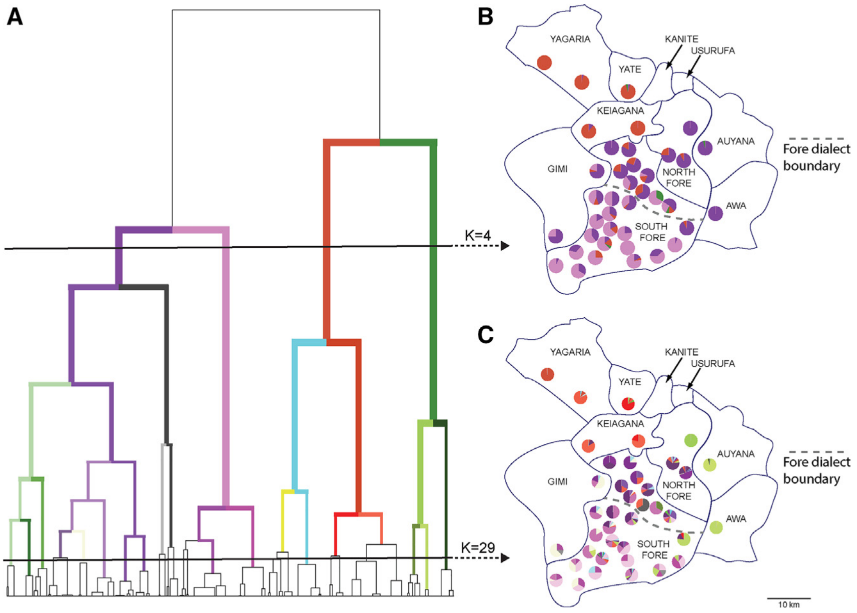

Figure 3. fineStructure clustering output taken from analysis of 943 EHPNG individuals from 21 linguistic groups
fineStructure dendrogram (A) with branches highlighted to match clustering of individuals for village sampling locations when K = 4 (B) and K = 29 (C).

We also explored the relationship between EHPNG to other PNG populations by performing a ChromoPainter/fineStructure analysis on a joint dataset containing the 943 EHPNG individuals in the village analysis dataset and publicly available data from 350 individuals sampled from other PNG regions. Visual inspection of the ChromoPainter heatmap and corresponding fineStructure tree (Figure S8) showed differential patterns of copying between the different EHPNG populations in relation to non-EHPNG populations. For example, individuals originating from northwest EHPNG (individuals who are in EHPNG cluster 10 in the village analysis dataset K = 12) clustered closely with individuals from the neighboring Chimbu population, which is administratively in a region neighboring EHPNG to the west. Also, Anga and Pawaians populations had markedly different patterns of copying in this analysis compared to other EHPNG clusters and to each other (Figure S8), with both groups clustering more closely with non-EHPNG populations including non-Highland populations (Figure S9). 11 individuals in the village analysis ChromoPainter and fineStructure analysis who appeared as recent long-distance migrants from other EHPNG groups in this analysis clustered with other Highland populations entirely, possibly reflecting inter-region migration. All of these individuals were born in the 1960s or later (after European contact) and included 2 individual outliers identified in PCA (Figure 1B).

To control for factors such as unequal sample size and incomplete lineage sorting that ChromoPainter does not directly address, we used SOURCEFIND to infer the proportion of DNA for which each of the 12 EHPNG clusters shares most recent ancestry with a set of other "surrogate" groups meant to represent ancestral sources of EHPNG populations. For these surrogate groups, we used the 10 geographically labeled populations from non-EHPNG regions as defined in a previous study and the 12 EHPNG populations defined as fineStructure clusters in the village analysis fineStructure analysis (Figures S5 and S6; Table S6). Our SOURCEFIND analysis allowed each EHPNG population to select ancestral sources from the 11 other EHPNG populations and the 10 non-EHPNG populatiopns (Table S7). Results from SOURCEFIND were consistent with fineStructure clustering, showing the same distinct relationships for Anga, Pawaian, and northwestern EHPNG clusters to external groups (Figure S9). For each of these groups, the largest ancestry sources (contributing >5%) were populations outside of EHPNG. Anga and Pawaian populations derived the majority of their ancestry from non-Highland sources (Anga 92% and Pawaian 82.5%). For all other EHPNG populations, nearest neighbors within EHPNG were the major contributing source.

Investigation of sex-biased migration informed by population structure

Clustering of individuals from different ethno-linguistic groups in fineStructure, ADMIXTURE, and PCA analyses (e.g., Figure 1) may reflect individual migrants within EHPNG. Assuming this is the case, we investigated whether there is evidence for sex-biased migration into kuru-affected areas. To do so, we inferred migrants based on the fineStructure clustering (and not PCA as per Figure 1B due to sample size biases in this dataset) of the village analysis dataset (see material and methods for details) and compared the number of such female and male migrants in three areas with differing kuru incidence rates (high, medium, and zero/low) to the number of females and males among non-migrants in the same areas (Table 1). We observed a higher proportion of females among migrants relative to among non-migrants in each kuru incidence classification, consistent with patrilocal practices, but we note that the difference in proportion of females between migrants and non-migrants was significant only in the high kuru incidence area (chi-squared test $p = 0.0145$) and not in the medium and low/zero kuru incidence areas (chi-squared test $p = 0.130$ and 0.067, respectively). However, when using a logistic regression that jointly models all individuals to test how the probability of being a migrant depends on sex and kuru incidence, we did not find evidence that the effect of sex differs

Table 1. Summary of migrant assignments and test of sex bias

Kuru disease exposure	Male non-migrants	Female non-migrants	Proportion of females among non-migrants	Male migrants	Female migrants	Proportion of females among migrants	p value
**High**	92	137	0.598	10	37	0.787	0.0145
**Medium**	67	95	0.586	11	28	0.718	0.130
**Zero/low**	180	166	0.480	16	27	0.628	0.0669

significantly between the three kuru exposure groups ($p = 0.7863$).

Discussion

We have presented an in-depth study of the population structure of a remote highland region of PNG, based on a dataset that includes most of the linguistic groups and extensive sampling by village, affording a level of resolution that has not previously been possible. We show that despite covering an area of only 11,157 km^2, roughly the size of the island of Jamaica, the genetic differentiation between linguistic groups in the EHPNG region is strikingly high (maximum $F_{ST} = 0.066$, average $F_{ST} = 0.024$, average distance 45 km). This differentiation is comparable to that previously reported for the entire PNG Highlands region,[19] of which EHPNG is only a small sub-region. Furthermore, our analyses reveal the presence of complex population structure even at dialect and village level. While of interest for understanding the origins of modern populations, these findings also provide the background for study of the genetic impact of the large-scale prion disease epidemic, kuru.

A key question is what factors have contributed to such strong population structure in such a small region. One possible factor is that while the Western Highlands had optimal conditions for taro cultivation, which originated there, whereas the conditions were suboptimal in EHPNG.[10] Indeed, some groups in EHPNG have been described as having been "proto-agriculturalists" retaining elements of hunter-gatherer subsistence in their lifestyles.[10] Analogous to other hunter-gatherer groups, most EHPNG linguistic groups have greatly reduced population densities compared to other highland regions[1] and lower historical effective population sizes.[19] These reduced population densities have likely led to increased effects of genetic drift between groups.

In addition to geography, overall broad-scale population structure correlates with linguistics. For example, the best estimated linguistic tree for these groups analyzed shows the Pawaian and Anga as outgroups (Figure 1C), echoing the genetic analysis. We even see examples of fine-scale parallels between linguistic and genetic differences in the Fore dialect groups. However, the correlation between linguistic groupings and geographical regions makes it difficult to disentangle the relative role of these two factors. When more linguistic data become available for these populations in the future, approaches that quantitatively explore these relationships will add greatly to our understanding of these dynamics.

The colonial era definition of linguistic groups, which has been used in previous genetic studies, does not always satisfactorily describe population structure in the region. For example, linguistic groups are barely distinguishable from one another genetically in the northwest of EHPNG. This relative homogeneity may be due to more intensely practised agriculture and higher population densities in this sub-region,[35] where the geography is different, with wide valleys in contrast to the highly dissected terrain in the southeast. Furthermore, in the village-level analyses of the Fore, we found genetic affinity of some villages to be closer to non-Fore groups. This observation is not completely surprising, as it is well understood that the clan structure of political, economic, and social unions that comprised the pre-colonial landscape in EHPNG often spanned linguistic group boundaries. This observed signature (for example as observed with Ilesa in the South Fore, Figure 3) could also be the result of a village founding event when a whole village is uprooted (e.g., due to conflict) and moves considerable distance to new territory, with members acquiring the language of their new residence. Such founding events in the past have been observed in the anthropological record within EHPNG.[12]

Several analyses revealed the presence of recent long-distance migration in the region. For instance, we found three potential migrants from the Fore linguistic group into the Pawaian (Figure 1A). One individual moved to the Pawaian linguistic area after marrying into a family there. However, marriages across the Fore/Pawaian divide are believed to be very rare or possibly nonexistent in pre-European-contact times, due to the considerable barriers of endemic warfare and extreme terrain. Consistent with this we found that all three observed migrants were born after European contact (although these individuals may be the descendants of migrants), which resulted in a cessation of warfare and the development of transport infrastructure which may have facilitated these movements. Hence it is possible that such long-distance migration is a recent phenomenon, consistent with the two groups being so genetically distant.

A final example demonstrating that analysis of population structure using linguistic group labels is not fully satisfactory was where we observed a clear resolution of village differences based on South Fore dialect spoken within the

linguistic group (Figure 3C). This suggests that pooling all South Fore into a single population may not adequately capture population structure, though in this case the small genetic difference does correlate with a small linguistic one. Certainly, it reveals the dynamic and ongoing processes of cultural and demographic change that has been unfolding in the region. Given the small genetic differences between the fineStructure clusters that represent the two dialect groups, the small geographic differences between different dialect villages, and the fact that marital exchange and migration was known to occur between villages across the dialect divide, it is likely that such a split was recent in origin. This echoes oral origin histories held among the Fore that details the expansion and fragmentation of the Fore people into the three distinct dialect groups.

One of the population structure patterns that very clearly follows the linguistic groupings is that the Anga and Pawaian linguistic groups appear highly genetically distant not only from all other groups, but also from each other. Clusters comprising these groups had highest genetic similarity with groups outside of EHPNG, rather than EHPNG neighbors, in contrast to all other EHPNG clusters except a cluster of northwest individuals (EHPNG cluster 10) who were genetically related to the neighboring Chimbu. In the case of the Pawaian, the inferred closest ancestry source was the southern Kiwai, a coastal population more than 300 km from EHPNG. Interestingly, the Pawaian linguistic group is known to live semi-nomadically in forests at lower elevations and lower population densities than the rest of the region,[1] which may in itself explain why they have ended up somewhat genetically distinct from the rest of the EHPNG groups. The oral histories held by the Pawaian speak of originating from coastal regions and undertaking long migrations through uninhabited forest regions.[26]

Our results do not support significant genetic influence on EHPNG populations other than the Anga and Pawaian from outside of the PNG highlands. In particular, unlike in many other regions of the world that have been colonized by countries with people of European descent,[36] we found no clear signs of European ancestry in EHPNG individuals (Figure S5). And while we did in our ADMIXTURE analyses observe some signatures that are consistent with a few individuals having a small amount of admixture with people of East Asian ancestry, this could just as well be caused by other PNG populations not being represented in those analyses.[19] Our findings of support previously suggested population histories[1,19] with an expansion of groups ("neolithic expansion") emanating from the Western Highlands as a result of the development of taro agriculture and displacement of previous groups that lived there, possibly ancestral to the Anga who have greatly distinct ancestry profiles in our analyses and who now live in the southern fringes of the region. In fact "Anga-like" artifacts, believed to be ancient, have been found as far north as in the Kamano linguistic group, reflecting a more widely dispersed settlement in the region.[1]

Our observation of recent migrant individuals in multiple analyses allowed the examination of the impact of kuru on migration dynamics in the region. While we observed a higher proportion of females among migrants relative to non-migrants throughout EHPNG, likely due to the general practice of patrilocality, the largest and only significant skew toward female migrants was observed in areas of high kuru incidence. This observation is consistent with accounts from the region that notes during the epidemic the near absence of adult women in villages with high kuru incidence.[24] Men would frequently marry multiple times as a result of their previous wives dying from kuru, and strains were also placed on communities as a result of increased child care burden.[23] Thus it seems plausible that the need to replace lost adult women and mitigate the impact of kuru on Fore society could have led to an excessive inflow of recent female migrants into villages with high kuru incidence. However, we note that previously the opposite has been reported: that kuru led to a decrease or even a complete cessation of intermarriages between the Fore and neighboring communities, because those communities linked kuru to sorcery, which made them fearful of the Fore.[37] In our data, we observed no evidence either for less overall migration into areas with high kuru incidence or for a stop of patrilocal practices in these areas. On the contrary, we observed a significant bias toward females among migrants into high kuru incidence areas. Moreover, the observed difference in the proportion of females among migrants versus migrants is ~25% higher in the "high" incidence kuru areas relative to the "zero/low" kuru incidence areas. While this difference was not statistically significant using the data here, it may reflect kuru causing a sex bias beyond that driven by patrilocal practices. We note that our approach considers only genetically distinct individuals to be migrants. This means we are also counting migrants that came from greater distances away than would have typically been the case for marital exchange. Hence, it is possible that bachelors within highly kuru-affected communities have sought wives from further afield than usual due to the lack of availability of potential wives more locally and that this led to the observed sex-bias in migration in the high kuru incidence areas. However, additional data and analyses are necessary to validate this. What our current data and analyses do suggest is that there was sex-biased migration into the high-kuru areas despite documented fears and strains placed on communities as a result of kuru.

In summary, our results suggest that the observed population structure is not driven by admixture from outside the highland PNG region, which is consistent with the historical record of the EHPNG region being isolated until recently. Also, while the population structure does to some extent mimic the linguistic groupings in the area, we observe several patterns of population structure that

suggest that the different linguistic groups are not entirely genetically distinct and isolated from each other. This is consistent with previous knowledge of clans playing a key role in the cultural grouping in the area and of the presence of cultural features that aided possible migration between neighboring linguistic groups in the region. Finally, we observed signs that long-distance migration has taken place, likely in more recent times. Importantly this, in combination with the understanding of population structure, has permitted an analysis of sex-biased flows of migration that are likely to have been impacted by kuru. This highlights that it is essential to understand the population structure of a region prior to attempting to investigate hypotheses regarding the impact of epidemics on affected populations.

The population structure of EHPNG reveals a complex multi-layered set of factors that have caused high population differentiation, likely including both geographic and cultural factors. Furthermore, it suggests that the current population structure may still be evolving. Overall our results demonstrate that simplistic descriptions of the population structure in regions like EHPNG based on linguistic groupings presumed to be static are likely to neglect the far richer texture of dynamic forces and history that has shaped communities.

Data and code availability

There are restrictions to the availability of the EHPNG genetic datasets due to the sensitive nature of these datasets in accordance with ongoing ethics and data sharing agreement between PNGIMR and UCL Institute of Prion Diseases.

Supplemental information

Supplemental information can be found online at https://doi.org/10.1016/j.ajhg.2024.02.011.

Acknowledgments

We acknowledge the help of the Eastern Highlands Province communities and the many individuals who have made this study possible. We thank the kuru project field staff of the Papua New Guinea Institute of Medical Research for their hard work on this study. These studies were supported by funding from the UK Medical Research Council (L.Q., J.W., T.C., H.H., J.C., and S.M.), the European Research Council (L.Q. and I.M.) (ERC-2018-STG-804679), and the Wellcome Trust and Royal Society (G.H., 098386/Z/12/Z and 224575/Z/21/Z).

Declaration of interests

The authors declare no competing interests.

Received: November 23, 2022
Accepted: February 20, 2024
Published: March 19, 2024

References

1. Gajdusek, D.C., and Alpers, M. (1972). Genetic studies in relation of kuru. I. Cultural, historical, and demographic background. Am. J. Hum. Genet. *24* (*Suppl*), 1–38.
2. Ballard, C. (2010). Watching First Contact. J. Pac. Hist. *45*, 21–36.
3. Feil, D.K. (1986). A Social Anthropologist's View of Papua New Guinea Highlands Prehistory. Am. Anthropol. *88*, 623–636.
4. Alpers, M.P. (2008). Review. The epidemiology of kuru: monitoring the epidemic from its peak to its end. Philos. Trans. R. Soc. Lond. B Biol. Sci. *363*, 3707–3713.
5. Nelson, H. (2000). Liberation: The End of Australian Rule in Papua New Guinea. J. Pac. Hist. *35*, 269–280.
6. Whitfield, J.T., Pako, W.H., Collinge, J., and Alpers, M.P. (2008). Mortuary rites of the South Fore and kuru. Philos. Trans. R. Soc. Lond. B Biol. Sci. *363*, 3721–3724.
7. Whitfield, J.T., Pako, W.H., and Alpers, M.P. (2015). Metaphysical personhood and traditional South Fore mortuary rites. jso. *141*, 303–321.
8. Radford, A.J., and Radford, R. (2010). Some Sources of Stone Axe Adze Heads in the Eastern Highlands of New Guinea and the Trade Routes Along Which They Travelled. Mankind *12*, 241–246.
9. Foley, W.A. (2000). The Languages of New Guinea. Annu. Rev. Anthropol. *29*, 357–404.
10. Feil, D.K. (1995). The Evolution of Highland Papua New Guinea Societies: A Reappraisal. Bijdr. taal. land. volkenkd. *151*, 23–43.
11. Liberski, P.P., and Brown, P. (2004). Kuru: a half-opened window onto the landscape of neurodegenerative diseases. Folia Neuropathol. *42*, 3–14.
12. Mandeville, E. (1979). Agnation, Affinity and Migration Among the Kamano of the New Guinea Highlands. Man *14*, 105–123.
13. Anas, M. (1960). The Highlands of Australian New Guinea. Geogr. Rev. *50*, 467–490.
14. Watson, J.B. (1970). Society as Organized Flow: The Tairora Case. SW. J. Anthropol. *26*, 107–124.
15. Malaspinas, A.S., Westaway, M.C., Muller, C., Sousa, V.C., Lao, O., Alves, I., Bergström, A., Athanasiadis, G., Cheng, J.Y., Crawford, J.E., et al. (2016). A genomic history of Aboriginal Australia. Nature *538*, 207–214.
16. Redd, A.J., and Stoneking, M. (1999). Peopling of Sahul: mtDNA variation in aboriginal Australian and Papua New Guinean populations. Am. J. Hum. Genet. *65*, 808–828.
17. Jacobs, G.S., Hudjashov, G., Saag, L., Kusuma, P., Darusallam, C.C., Lawson, D.J., Mondal, M., Pagani, L., Ricaut, F.X., Stoneking, M., et al. (2019). Multiple Deeply Divergent Denisovan Ancestries in Papuans. Cell *177*, 1010–1021.e32.
18. Stoneking, M., Jorde, L.B., Bhatia, K., and Wilson, A.C. (1990). Geographic variation in human mitochondrial DNA from Papua New Guinea. Genetics *124*, 717–733.
19. Bergström, A., Oppenheimer, S.J., Mentzer, A.J., Auckland, K., Robson, K., Attenborough, R., Alpers, M.P., Koki, G., Pomat, W., Siba, P., et al. (2017). A Neolithic expansion, but strong genetic structure, in the independent history of New Guinea. Science *357*, 1160–1163.
20. Collinge, J., Whitfield, J., McKintosh, E., Beck, J., Mead, S., Thomas, D.J., and Alpers, M.P. (2006). Kuru in the 21st century–an acquired human prion disease with very long incubation periods. Lancet *367*, 2068–2074.

21. Collinge, J. (2001). Prion diseases of humans and animals: their causes and molecular basis. Annu. Rev. Neurosci. *24*, 519–550.
22. Gajdusek, D.C., and Zigas, V. (1959). Kuru; clinical, pathological and epidemiological study of an acute progressive degenerative disease of the central nervous system among natives of the Eastern Highlands of New Guinea. Am. J. Med. *26*, 442–469.
23. Lindenbaum, S. (2008). Review. Understanding kuru: the contribution of anthropology and medicine. Philos. Trans. R. Soc. Lond. B Biol. Sci. *363*, 3715–3720.
24. Mead, S., Whitfield, J., Poulter, M., Shah, P., Uphill, J., Campbell, T., Al-Dujaily, H., Hummerich, H., Beck, J., Mein, C.A., et al. (2009). A novel protective prion protein variant that colocalizes with kuru exposure. N. Engl. J. Med. *361*, 2056–2065.
25. Wiesenfeld, S.L., and Gajdusek, C. (1976). Genetic structure and heterozygosity in the kuru region, Eastern highlands of New Guinea. Am. J. Phys. Anthropol. *45*, 177–190.
26. Bhatia, K., Prasad, M.L., Barnish, G., and Koki, G. (1988). Antigen and haplotype frequencies at three human leucocyte antigen loci (HLA-A, -B, -C) in the Pawaia of Papua New Guinea. Am. J. Phys. Anthropol. *75*, 329–340.
27. Clarke, L., Fairley, S., Zheng-Bradley, X., Streeter, I., Perry, E., Lowy, E., Tassé, A.M., and Flicek, P. (2017). The international Genome sample resource (IGSR): A worldwide collection of genome variation incorporating the 1000 Genomes Project data. Nucleic Acids Res. *45*, D854–D859.
28. Purcell, S., Neale, B., Todd-Brown, K., Thomas, L., Ferreira, M.A.R., Bender, D., Maller, J., Sklar, P., de Bakker, P.I.W., Daly, M.J., and Sham, P.C. (2007). PLINK: a tool set for whole-genome association and population-based linkage analyses. Am. J. Hum. Genet. *81*, 559–575.
29. Lawson, D.J., Hellenthal, G., Myers, S., and Falush, D. (2012). Inference of population structure using dense haplotype data. PLoS Genet. *8*, e1002453.
30. Conrad, D.F., and Hurles, M.E. (2007). The population genetics of structural variation. Nat. Genet. *39*, S30–S36.
31. Alexander, D.H., and Lange, K. (2011). Enhancements to the ADMIXTURE algorithm for individual ancestry estimation. BMC Bioinf. *12*, 246.
32. Delaneau, O., Marchini, J., and Zagury, J.F. (2011). A linear complexity phasing method for thousands of genomes. Nat. Methods *9*, 179–181.
33. Chacón-Duque, J.C., Adhikari, K., Fuentes-Guajardo, M., Mendoza-Revilla, J., Acuña-Alonzo, V., Barquera, R., Quinto-Sánchez, M., Gómez-Valdés, J., Everardo Martínez, P., Villamil-Ramírez, H., et al. (2018). Latin Americans show wide-spread Converso ancestry and imprint of local Native ancestry on physical appearance. Nat. Commun. *9*, 5388.
34. Konishi, T. (2015). Principal component analysis for designed experiments. BMC Bioinf. *16* (*Suppl 18*), S7.
35. Gregori, T.D., and Salisbury, R.F. (1964). From Stone to Steel: Economic Consequences of a Technological Change in New Guinea. Technol. Cult. *5*, 272–274.
36. Hellenthal, G., Busby, G.B.J., Band, G., Wilson, J.F., Capelli, C., Falush, D., and Myers, S. (2014). A genetic atlas of human admixture history. Science *343*, 747–751.
37. Klitzman, R. (1999). Sorcery and science: responses to kuru and other epidemics. West. J. Med. *171*, 204–206.

The American Journal of Human Genetics, Volume *111*

Supplemental information

Population structure and migration in the Eastern Highlands of Papua New Guinea, a region impacted by the kuru epidemic

Liam Quinn, Jerome Whitfield, Michael P. Alpers, Tracy Campbell, Holger Hummerich, William Pomat, Peter Siba, George Koki, Ida Moltke, John Collinge, Garrett Hellenthal, and Simon Mead

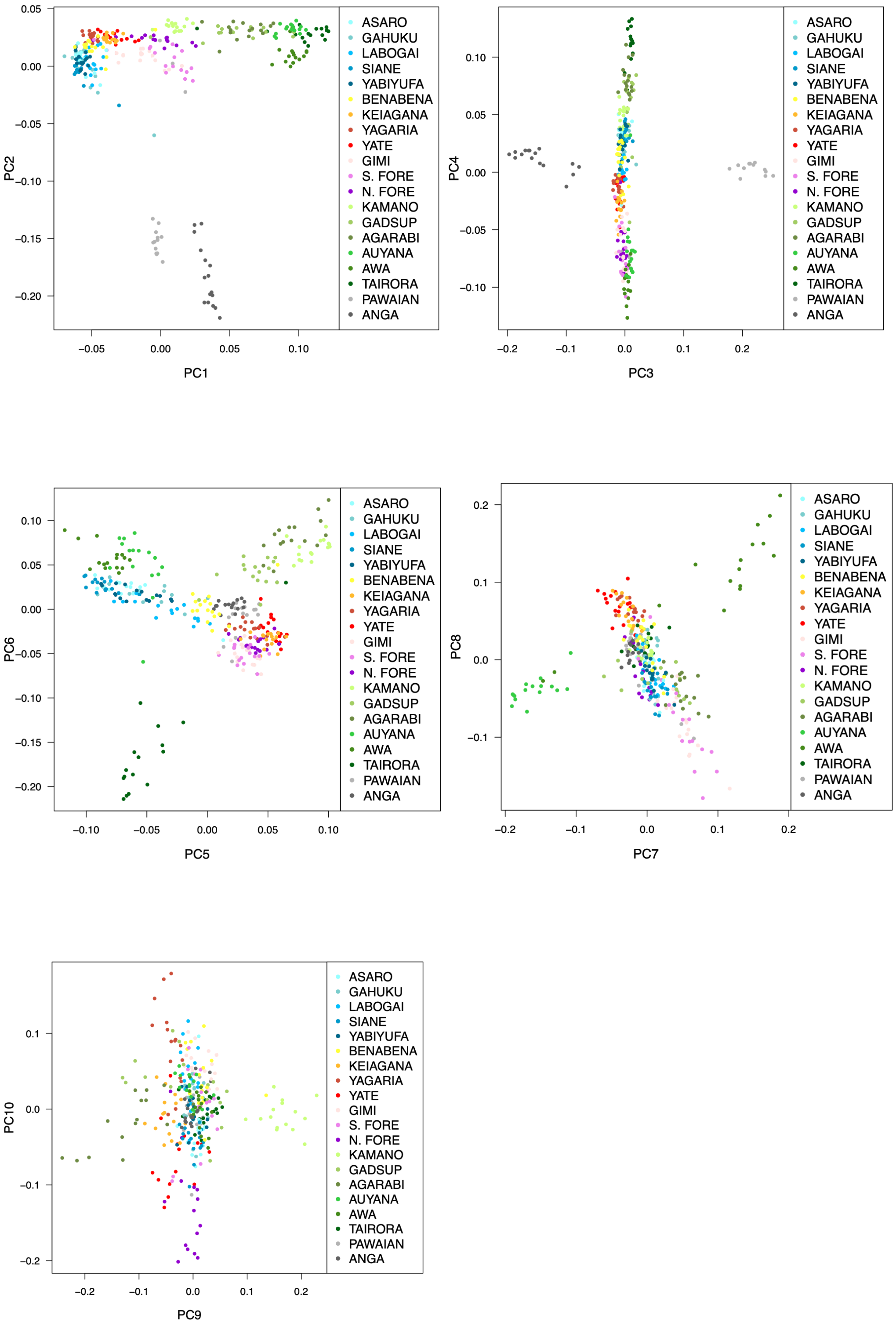

Figure S1 - Principal Components 1-10 of 320 individuals from 20 EHPNG linguistic groups

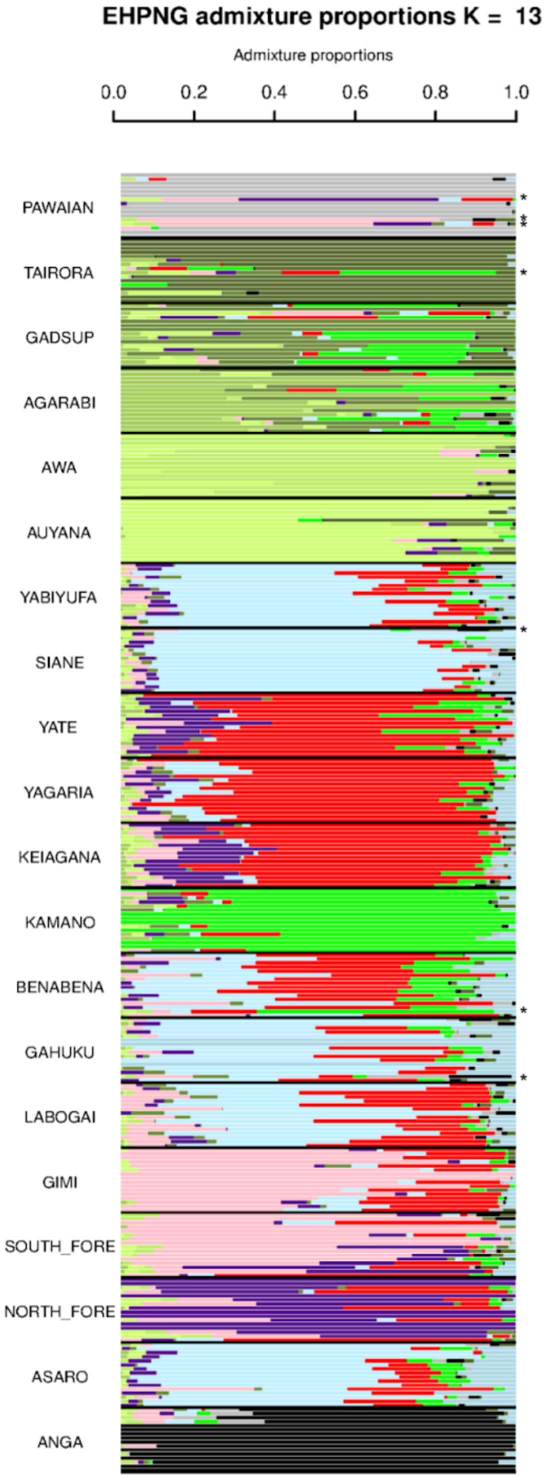
EHPNG admixture proportions K = 13
Admixture proportions
0.0
0.2
0.4
0.6
0.8
1.0
PAWAIAN
TAIRORA
GADSUP
AGARABI
AWA
AUYANA
YABIYUFA
SIANE
YATE
YAGARIA
KEIAGANA
KAMANO
BENABENA
GAHUKU
LABOGAI
GIMI
SOUTH_FORE
NORTH_FORE
ASARO
ANGA

Figure S2 - ADMIXTURE analysis of 320 individuals from 20 EHPNG linguistic groups for K=13. Individuals with * label in above ADMIXTURE profile are those individuals highlighted in Figure 1a and Figure 1b as potential migrants.

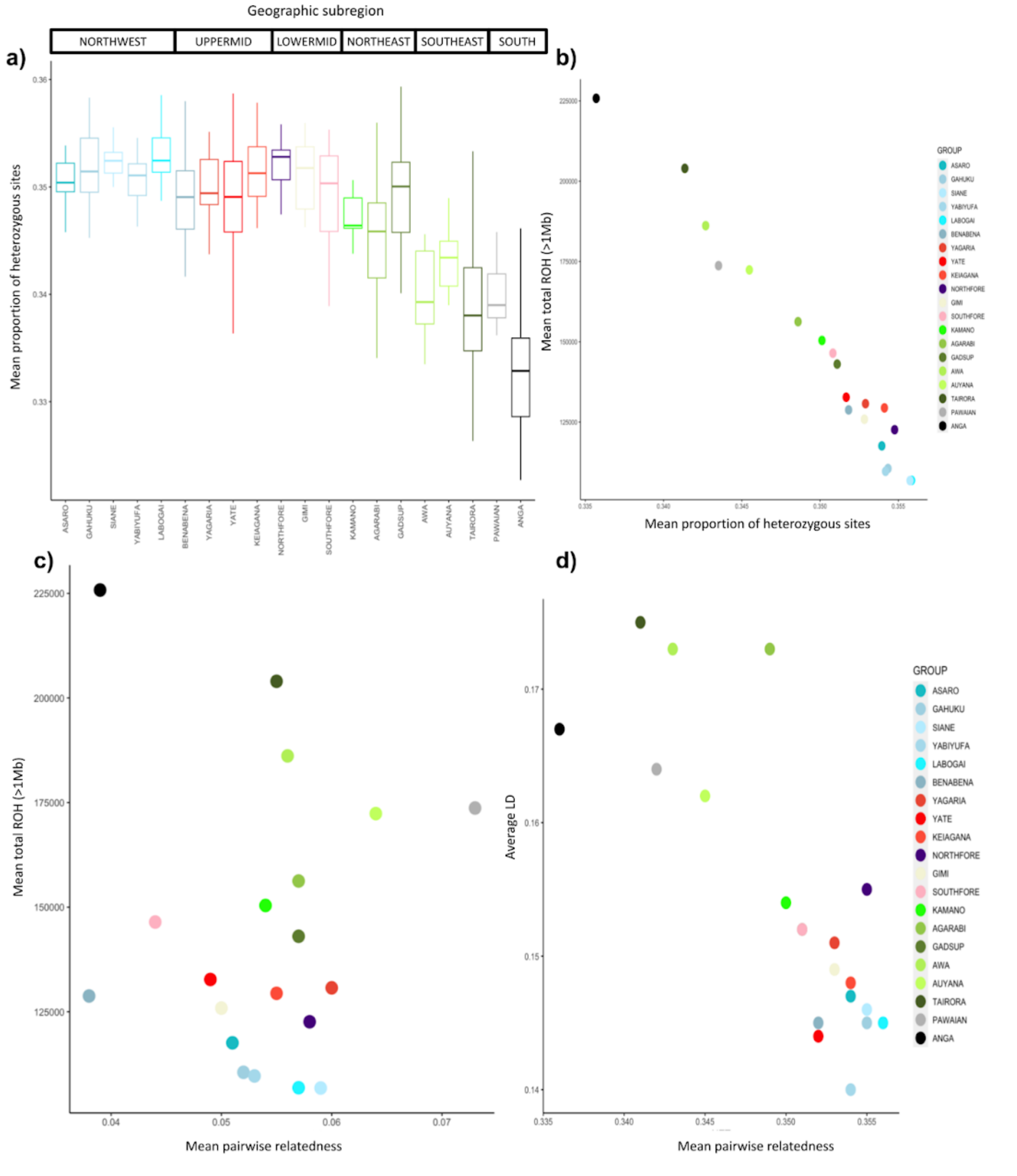

Figure S3 - a) Boxplot of mean proportion of heterozygous sites by geographic sublocation. b) Mean total length of ROH versus proportion of heterozygous sites. c) Mean total length of ROH versus mean pairwise relatedness. d) Mean average pairwise relatedness versus mean linkage disequilibrium. Plots are taken from analysis of *Linguistic Group Analysis* dataset.

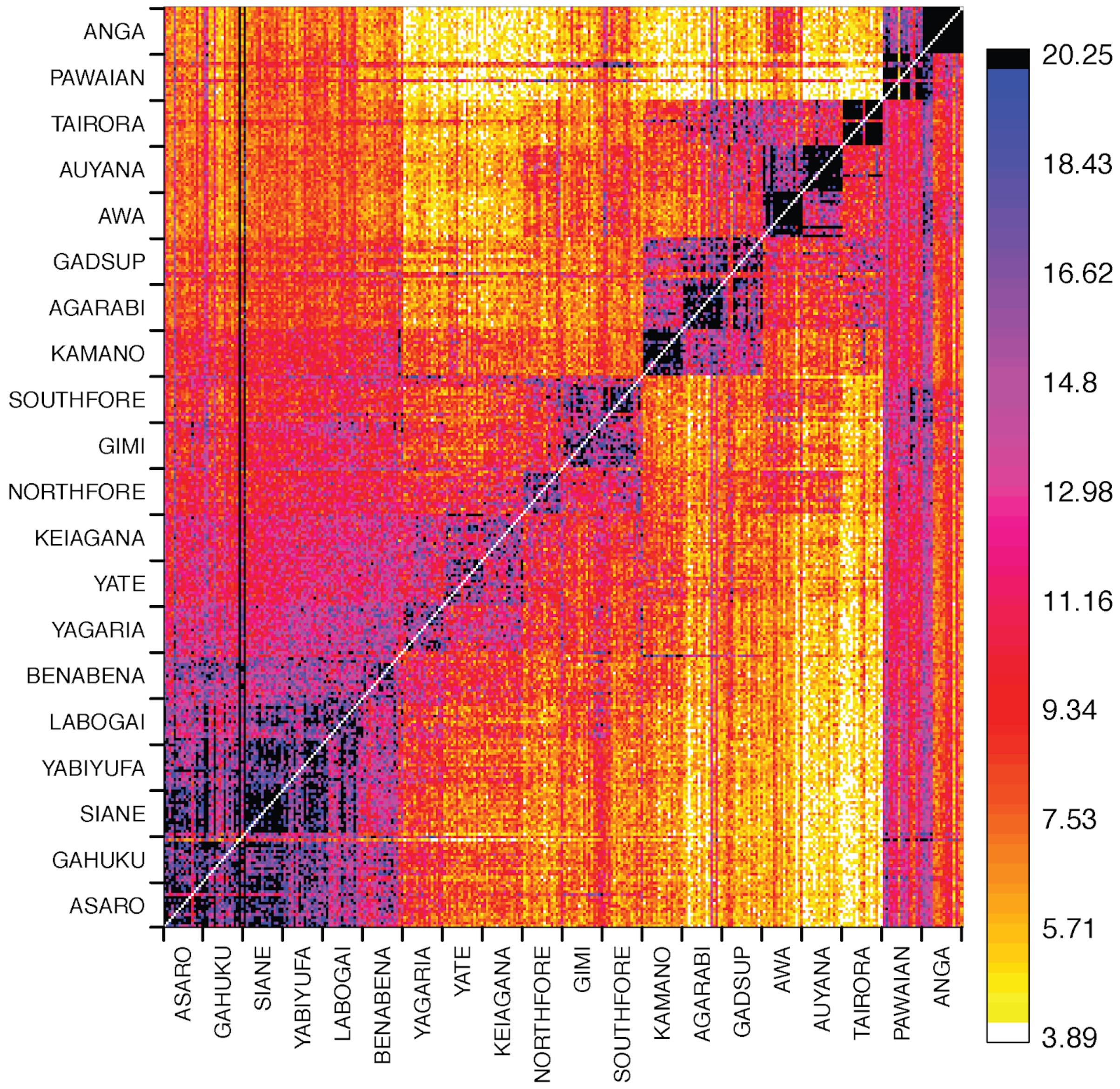

Figure S4 - CP heatmap of 320 individuals from 20 EHPNG linguistic groups.

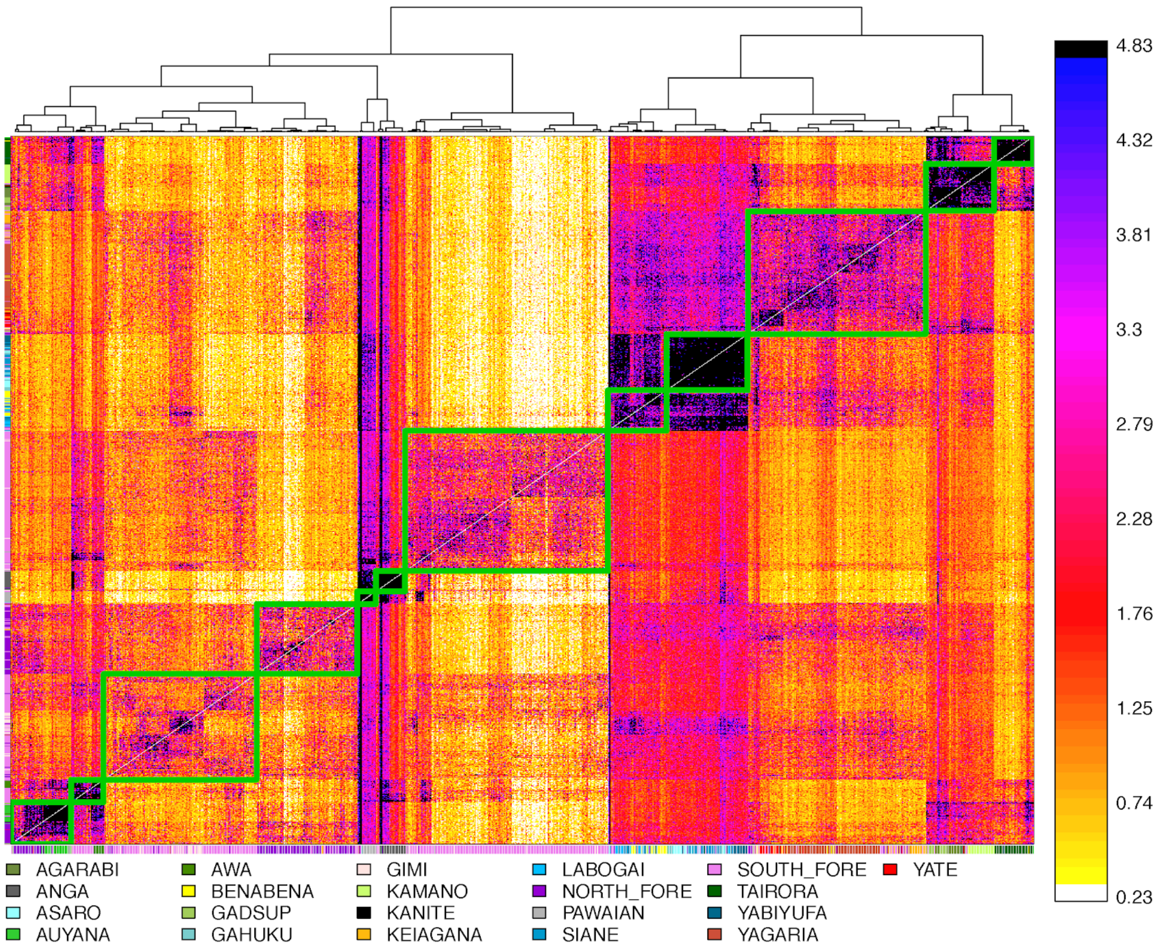

Figure S5 – CP heatmap of 943 individuals from 21 EHPNG Ethno-Linguistic Groups placed into 12 discrete clusters (green boxes on heatmap) as defined by FS.

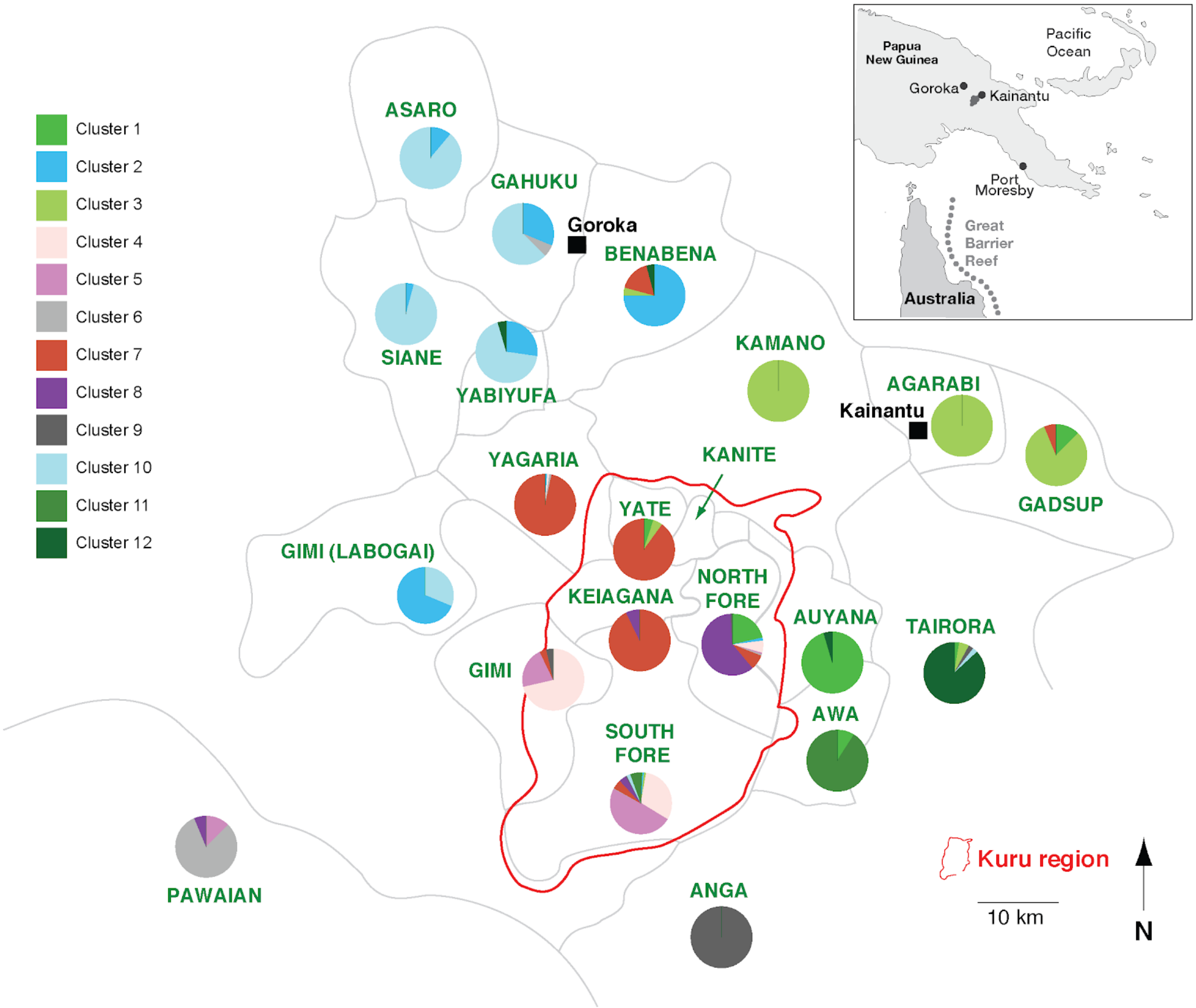

Figure S6 - Geographical representation of FS clustering (K=12) of individuals from *Village Analysis* CP output of Figure S5.

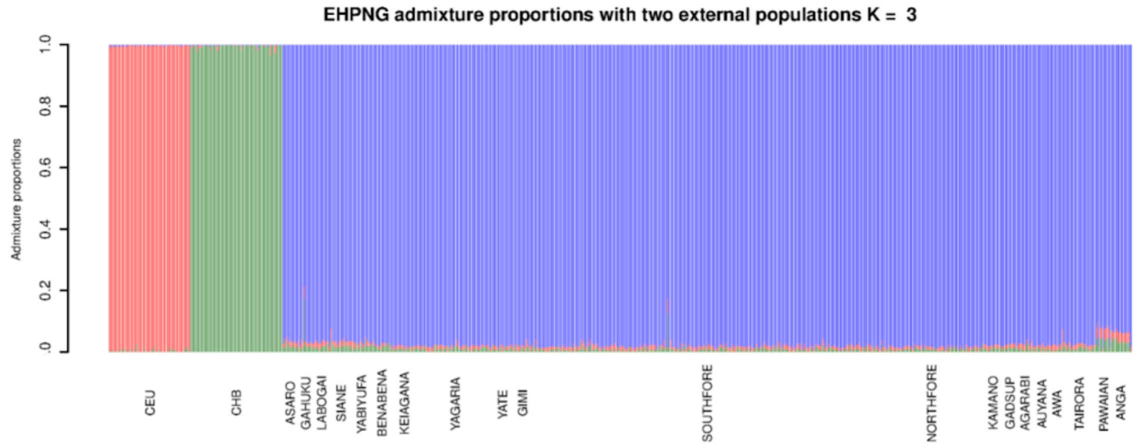

Figure S7 – ADMIXTURE analysis of 943 EHPNG individuals from 21 linguistic groups with 1000 genomes project populations CEU and YRI.

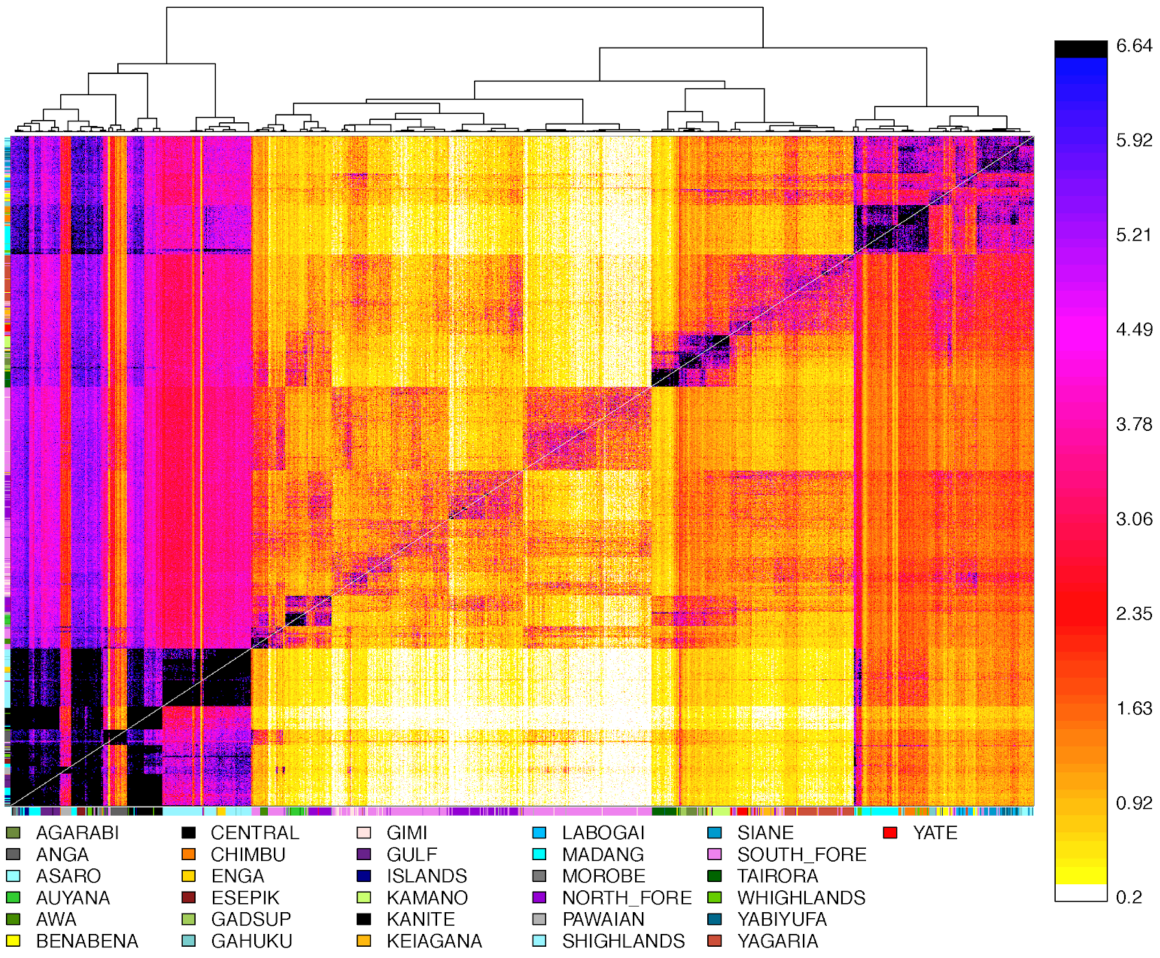

Figure S8 –CP heatmap of 1293 individuals, 943 from 21 EHPNG linguistic groups and 350 individuals from other PNG regions.

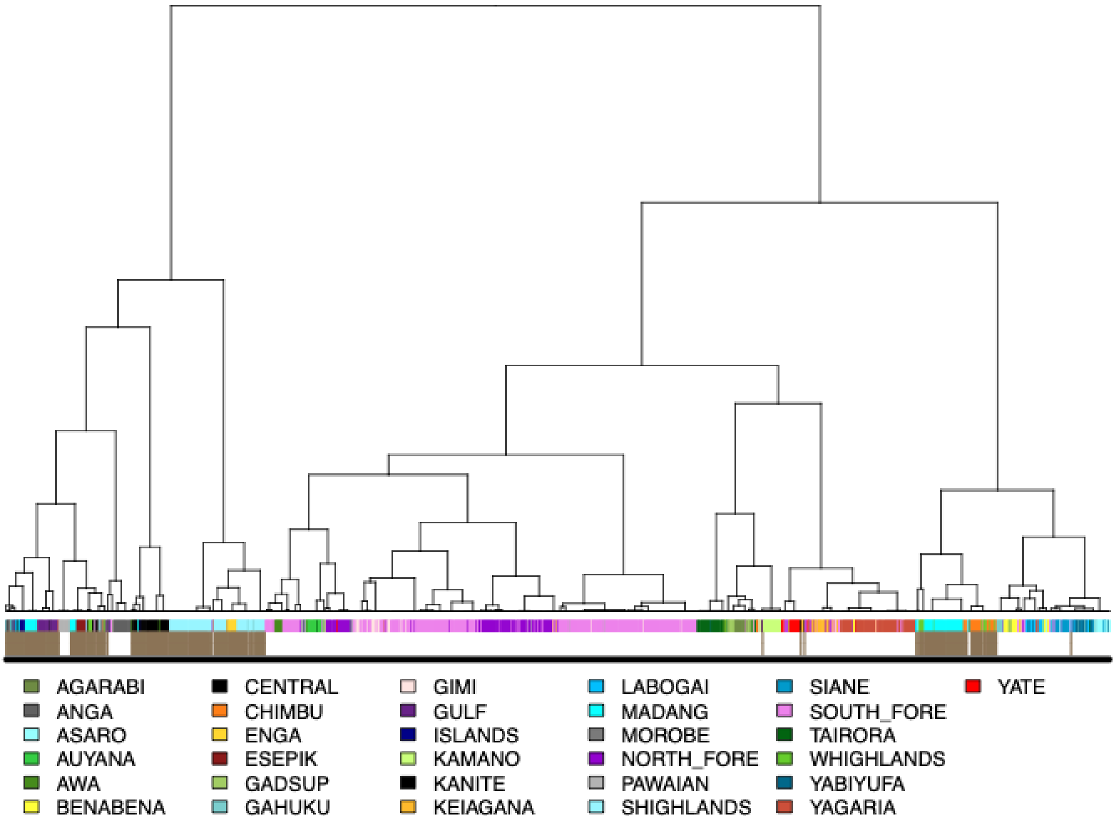

Figure S9 - FS Tree from CP analysis of 943 EPHNG individuals and 350 individuals from other PNG regions based on CP output (Figure S8). Light grey vertical lines connecting the horizontal black line to the linguistic group color labels highlight the 350 non EHPNG individuals. The majority of Individuals from Anga and Pawaian populations split from other EHPNG populations at K=2 of this tree with populations from other EHPNG regions. EHPNG Individuals from Siane, Gahuku, Asaro, Benabena and Yabiyufa linguistic groups (EHPNG Cluster 10 Figure S5, S6) in the north-west of the region split from remaining EHPNG populations at K=3 cluster with neighbouring non-EHPNG populations Chimbu and Madang.

POPULAITON	NUMBER OF SAMPLES	ORIGIN
EHPNG	1374	PRIMARY DATA
PNG	380	ACCESS AGREEMENT
ACB	95	1000 GENOMES
Altai	1	ANCIENT HOMININ
ASW	45	1000 GENOMES
Batwa	5	AFRICA
BEB	83	1000 GENOMES
CDX	82	1000 GENOMES
CEU	91	1000 GENOMES
CHB	103	1000 GENOMES
CHS	97	1000 GENOMES
CLM	93	1000 GENOMES
Colla	22	SOUTH AMERICA
Denisova	1	ANCIENT HOMININ
ESN	95	1000 GENOMES
FIN	99	1000 GENOMES
GB20	1	ANCIENT EUROPEAN
GBR	85	1000 GENOMES
GIH	96	1000 GENOMES
GWD	112	1000 GENOMES
IBS	107	1000 GENOMES
ITU	98	1000 GENOMES
JPT	104	1000 GENOMES
Khoesan_ColouredColesberg	20	AFRICA
Khoesan_ColouredWellington	20	AFRICA
Khoesan_GuiGhanaKgal	15	AFRICA
Khoesan_Juhoansi	18	AFRICA
Khoesan_Karretjie	20	AFRICA
Khoesan_Khomani	39	AFRICA
Khoesan_Khwe	17	AFRICA
Khoesan_Nama	20	AFRICA
Khoesan_SEBantu	20	AFRICA
Khoesan_SWBantu	12	AFRICA
Khoesan_Xun	19	AFRICA
KHV	98	1000 GENOMES

LBK	1	ANCIENT EUROPEAN
Loschbour	1	ANCIENT EUROPEAN
LWK	79	1000 GENOMES
MSL	69	1000 GENOMES
MXL	55	1000 GENOMES
PEL	76	1000 GENOMES
PJL	86	1000 GENOMES
PUR	104	1000 GENOMES
Pygmy_Baka_Cam	56	AFRICA
Pygmy_Baka_Gab	16	AFRICA
Pygmy_Bakiga	34	AFRICA
Pygmy_Batwa	27	AFRICA
Pygmy_Bongo_GabE	22	AFRICA
Pygmy_Bongo_GabS	24	AFRICA
Pygmy_Nzebi_Gab	20	AFRICA
Pygmy_Nzime_Cam	52	AFRICA
STU	96	1000 GENOMES
TSI	106	1000 GENOMES
Ust_Ishim	1	ANCIENT EUROPEAN
Wichi	19	SOUTH AMERICA
YRI	101	1000 GENOMES

Table S1 - Populations used in Phasing Panel.

Village breakdown of ‘Village dataset’

**LINGUISTIC GROUP** / VILLAGE	COUNT OF INDIVIDUALS
**AGARABI**	
KAINOA	17
**ANGA**	
BOIKO	3
DUNGWI	15
SIMBARI	2
**ASARO**	
GIMISEVI	18
**AUYANA**	
OIYANA	22
**AWA**	
TAUNA	11
**BENABENA**	
MATAUSA	24
**GADSUP**	
UKARUMPA	16
**GAHUKU**	
HORIPOKAVE	16
**GIMI**	
EMO	15
ETEVE	8
HAIYARU	1
UNKNOWN	4
**KAMANO**	
HOMORI	24
**KANITE**	
UNKNOWN	3
**KEIAGANA**	
HOGATERU	18
UWAMI	9
**LABOGAI**	
LOSAVE	19
**NORTHFORE**	
ANUMPA	76
AWANDE	21
KALU	16
OKAPA	1
UNKNOWN	11
**PAWAIAN**	
UNKNOWN	16
**SIANE**	

WAIFO	24
**SOUTHFORE**	
KETABI	1
AGAKAMATASA	12
AI	5
AMORA	3
AWAROSA	9
HIGITARU	1
ILESA	13
INTAMATASA	6
IVAKI	35
KALU	6
KAMATA	12
KAMIRA	16
KANIGITASA	23
KASARAI	3
KEIAKASA	2
KETABI	12
KUME	6
MENTILASA	9
MIARASA	9
MUGAIAMUTI	35
OMA-KASORU	3
PAITI	15
PUROSA-TAKAI	22
TAKAI	27
TAKARI	8
TAMOGAVISA	1
UMASA	6
UNKNOWN	23
URAI	4
WAISA	25
WANIKANTO	2
WANITABI	5
YAGAREBA	2
YAGUSA	1
YASUBI	4
**TAIRORA**	
BONTA	38
**YABIYUFA**	
HOIHATOBE	22
**YAGARIA**	
KIWURUGA	49
NUSAGUNA	38
**YATE**	

KAUNA	20
Grand Total	943

Table S2 - Table of village localities in the *Village Analysis* dataset of 943 individuals from 21 EHPNG linguistic groups.

Analysis	Figure	Original merge *	Number of sites	Filters	Number of individuals	Sites after filters
PCA	Figure 1b	Linguistic Group	699865	--geno 0.01 --remove Related inds --indep-pairwise 50 5 0.8 –maf 0.05	320	222812
ADMIXTURE	Figure S2	Linguistic Group	699865	--geno 0.01 --remove Related inds --indep-pairwise 50 5 0.8 –maf 0.05	320	222812
FST	Table S2	Linguistic Group	699865	--merge 1000 Genomes --geno 0.01 --remove Related inds	313	369464
ROH	Table S5, Figure S3	Linguistic Group	699865	--merge 1000 Genomes --geno 0.01 --remove Related inds –maf 0.05	313	365678
Proportion of heterozygous sites	Table S5, Figure S3	Linguistic Group	699865	--merge 1000 Genomes --geno 0.01 --remove Related inds –maf 0.05	313	365678
LD	Figure 2, Table S5	Linguistic Group	699865	--merge 1000 Genomes --geno 0.01 --remove Related inds --chr 22 --maf 0.05 (per population)	260	≈ 4,000 per population
CP/FS (1)	Figure 1a, Figure S4	Village Analysis	240774	--merge Phasing panel --geno 0.01 --remove Related inds	320	122663
CP/FS (2)	Figure 3, Figure S5, S6	Village Analysis	240774	--merge Phasing panel --geno 0.01 --remove Related inds	943	122663
CP/FS (3)	Figure S8, S9	Village Analysis	240774	--merge Phasing panel --geno 0.01 --remove Related inds	1293	122663
ADMIXTURE (2)	Figure S7	Village Analysis	240774	--merge 1000 Genomes --geno 0.01 --remove Related inds --indep-pairwise 50 5 0.4	943	77828
SOURCEFIND	Table S6 S7	Village Analysis	240774	--merge Phasing panel --geno 0.01 --remove Related inds	1293	122663

Table S3 - Table showing sites filters for each analysis.

Table S4 - Has been provided as a separate table.

F_{ST} analysis of 20 EHPNG linguistic groups after 7 outlier individuals identified in PCA were removed.

Population	Mean proportion of heterozygous sites	Mean total ROH (> 1Mb)	Average LD	Mean pairwise relatedness
Anga	0.336	226	0.167	0.039
Pawaian	0.342	179	0.164	0.073
Tairora	0.341	208	0.175	0.055
Auyana	0.345	172	0.162	0.064
Awa	0.343	186	0.173	0.056
Gadsup	0.351	143	0.152	0.057
Agarabi	0.349	156	0.173	0.057
Kamano	0.350	150	0.154	0.054
southFore	0.351	146	0.152	0.044
Gimi	0.353	126	0.149	0.05
northFore	0.355	123	0.155	0.058
Keiagana	0.354	129	0.148	0.055
Yate	0.352	133	0.144	0.049
Yagaria	0.353	131	0.151	0.06
BenaBena	0.352	130	0.145	0.038
Labogai	0.356	107	0.145	0.057
Yabiyufa	0.354	110	0.14	0.053
Siane	0.355	110	0.146	0.059
Gahuku	0.355	109	0.145	0.052
Asaro	0.354	118	0.147	0.051

Table S5 - Summary of genetic statistics for 20 EHPNG linguistic groups. Groups are ordered according to geography in the same way as in the F_{ST} table (Figure S4). Runs of Homozygosity (ROH) is the mean total length of long runs of homozygosity (>1Mb) per individual in each population.

	EHPNG FS Cluster *Village Analysis* dataset											
**LINGUISTIC GROUP**	**1**	**2**	**3**	**4**	**5**	**6**	**7**	**8**	**9**	**10**	**11**	**12**
**ANGA**	0	0	0	0	0	0	0	0	22	0	0	0
**PAWAIAN**	0	0	0	0	2	13	0	1	0	0	0	0
**TAIRORA**	1	0	2	0	0	0	0	0	1	1	0	33
**AUYANA**	21	0	0	0	0	0	0	0	0	0	0	1
**AWA**	1	0	0	0	0	0	0	0	0	0	10	0
**GADSUP**	2	0	13	0	0	0	1	0	0	0	0	0
**AGARABI**	0	0	17	0	0	0	0	0	0	0	0	0
**KAMANO**	0	0	24	0	0	0	0	0	0	0	0	0
**SOUTH FORE**	3	3	4	112	178	1	18	14	2	7	20	0
**GIMI**	0	0	0	20	6	0	1	0	1	0	0	0
**NORTH FORE**	27	2	0	8	1	1	10	76	1	0	0	0
**KANITE**	0	0	1	0	0	0	2	0	0	0	0	0
**KEIAGANA**	0	0	0	0	0	0	26	2	0	0	0	0
**YATE**	1	0	1	0	0	0	18	0	0	0	0	0
**YAGARIA**	0	1	0	1	0	1	84	0	0	0	0	0
**BENABENA**	0	18	1	0	0	0	4	0	0	0	0	1
**LABOGAI**	0	16	0	0	0	0	0	0	0	3	0	0
**YABIYUFA**	0	6	0	0	0	0	0	0	0	15	0	1
**SIANE**	0	1	0	0	0	0	0	0	0	23	0	0
**GAHUKU**	0	5	0	0	0	1	0	0	0	10	0	0
**ASARO**	0	2	0	0	0	0	0	0	0	16	0	0

Table S6 – Breakdown of FS clustering in *Village Analysis* dataset (n=943); K=12.

Table S7 is provided as a separate excel sheet
